# In-Depth Mass Spectrometry Study of Vanadium(IV) Complexes with Model Peptides

**DOI:** 10.1021/acs.inorgchem.4c02683

**Published:** 2024-09-12

**Authors:** Kira Küssner, Valeria Ugone, Daniele Sanna, Monika Cziferszky

**Affiliations:** † Institute for Pharmacy, Pharmaceutical Chemistry, Department of Chemistry and Pharmacy, University of Innsbruck, Innrain 80/82, Innsbruck A-6020, Austria; ‡ Consiglio Nazionale delle Ricerche, Istituto di Chimica Biomolecolare, Traversa La Crucca 3, Sassari 07040, Italy

## Abstract

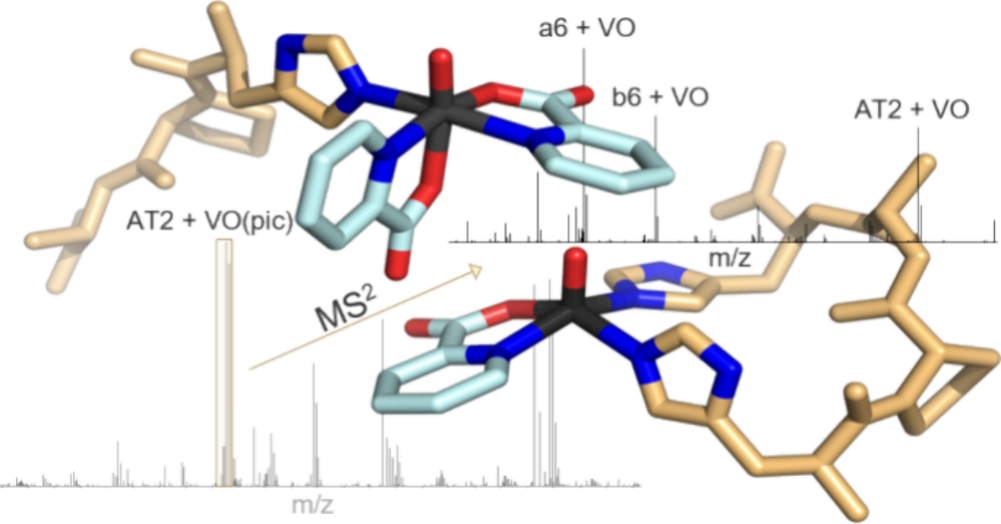

Investigating the speciation of vanadium complexes in the presence of potential biomolecular targets under physiological conditions remains challenging, and further experimental techniques are needed to better understand the mechanism of action of potential metallodrugs. The interaction of two model peptides (angiotensin I and angiotensin II) with three well-known oxidovanadium(IV) compounds with antidiabetic and/or anticancer activity, [V^IV^O(pic)_2_(H_2_O)], [V^IV^O(ma)_2_], and [V^IV^O(dhp)_2_] (where pic, ma, and dhp are picolinate, maltolate, and 1,2-dimethyl-3-hydroxy-4(1H)-pyridinonate anions, respectively), was investigated by ESI-MS/MS (electrospray ionization tandem mass spectrometry) and complemented by EPR (electron paramagnetic resonance) spectroscopy measurements and theoretical calculations at the DFT (density functional theory) level. The results demonstrated that vanadium–peptide bonds are preserved after HCD (higher energy collisional dissociation) fragmentation, allowing for the identification of binding sites through a detailed analysis of the fragmentation spectra. Angiotensin I (AT1) and angiotensin II (AT2) exhibited different coordination behaviors. AT1, with two His residues (His6, His9), prefers to form [AT1 + VOL] adducts with both histidine residues coordinated to the metal ion, while AT2, which has only His6, can bind the metal in a monodentate fashion, forming also [AT2 + VOL_2_] adducts. Insights from this study pave the way to ESI-MS/MS investigations of more complex systems, including target proteins and further development of vanadium-based drugs.

## Abstract 2

Top-down ESI-MS/MS (electrospray ionization tandem mass spectrometry) methods were employed to investigate the molecular interactions between angiotensin I and angiotensin II with three oxidovanadium(IV) complexes [VOL_2_]. The findings, combined with EPR (electron paramagnetic resonance) measurements and DFT (density functional theory) calculations, contribute to elucidating the coordination behavior of the two model peptides and give detailed information about binding preferences toward [V^IV^O]^2+^ species.

## Introduction

Vanadium compounds and their application as potential metallopharmaceuticals have been extensively explored over the past 40 years.^1−10^ While their antidiabetic effect has been recognized since the late 19th century,^11^ a variety of other pharmacological benefits, including antiparasitic,^12^ antibacterial,^13^ anti-HIV,^4^ and antitumor^5,8,14,15^ properties, of vanadium salts or complexes have been discovered in recent decades. This versatility can be attributed to the wide range of pharmacokinetic equilibriums within the organism and the flexible coordination chemistry.^15^ Of the various possible oxidation states in which vanadium can exist, +III to +V are considered as physiologically relevant and constantly interconvert dependent on the presence of oxidizing or reducing biomolecules.^1,6,8,16,17^ The attention within the scientific community increased significantly since pioneering studies on inorganic^18−20^ and ligand-coordinated^21^ vanadium compounds revealed important insights into their pharmacological mode of action.^7^ Pentavalent vanadate inhibits protein tyrosine phosphatases (PTPs) due to its structural similarity to phosphate. Inhibition of PTP1B enhances the insulin activity and is considered as the most dominant pharmacological effect.^2,15,22^ The poor gastrointestinal absorption of inorganic vanadium, which renders their oral administration dose close to the toxic level,^4,21^ could be improved by introducing hydrophobic organic ligands to adjust the hydrophilic/lipophilic balance and thus tailor their bioavailability.^1,23^ The benchmark vanadium complex tested as an antidiabetic agent, [V^IV^O(ma)_2_] (ma = maltolate), was reported to increase the potency of vanadium inorganic salts by about 50%.^21^ This discovery encouraged the development of a variety of [V^IV^OL_2_] complexes, were L represents a monoanionic bidentate ligand.^2,3,6,10,15,16^ Promising candidates from this class of oxidovanadium(IV) complexes are coordinated by pyridinone-derived ligands, like [V^IV^O(dhp)_2_] (dhp = dimethyl-3-hydroxy-4(1H)-pyridinonate) and [V^IV^O(pic)_2_] (pic = picolinate).^23,24^ The strong coordination especially of the dhp ligand leads to high biological stability^24^ and increased efficacy as an antidiabetic and anticancer agent.^10^ Despite their potential benefits, global pharmaceutical companies have shown little interest in vanadium compounds. This is due to the assumption that all vanadium complex derivatives undergo rapid hydrolysis with water or biomolecules, resulting in the exclusive formation of oxidovanadium(IV) or vanadate ions, complexed by bioligands.^22^ Thus, new screening methods are required to clarify the pharmacokinetic behavior, such as transportation pathways, physiological biotransformation, and binding site preferences with various biological counterparts. X-ray diffraction (XRD) and electron paramagnetic resonance (EPR) measurements can provide important insights into structural features. However, they are not able to completely characterize the biospeciation.^7^

Electrospray ionization mass spectrometry (ESI-MS) was first considered in 1999 to study adduct formation between cisplatin and ubiquitin as a model peptide.^25^ Since then, MS-based metallomics-studies have become an essential and indispensable tool to gain information about the number and stoichiometry of the adducts and their composition.^7,26,27^ Up to now, ESI-MS techniques have been mainly applied to more inert metal complexes of the second and third transition series, in which proteins bind with a coordination bond.^27^ Nevertheless, in recent years, different studies have appeared in literature in the field of vanadium chemistry, where ESI-MS was used to study the interaction with model proteins such as lysozyme,^17,28−31^ ubiquitin,^7,17,29,30^ myoglobin,^29,32^ cytochrome c,^10,28^ and ribonuclease A.^33^ In particular, ESI-MS enabled gaining information on the number and stoichiometry of the metal–protein adducts formed in solution at low concentrations (μM), while integrated EPR/computational studies facilitated the identification of potential binding sites. In general, it was observed that proteins can coordinate [V^IV^OL_2_] complexes in a monodentate or multidentate manner, depending on the stability and geometry of the complex. When the complex is stable and exists in solution as *cis*-[V^IV^OL_2_(H_2_O)], [VOL_2_]_*n*_-protein adducts are formed after the replacement of the equatorial water ligand by a His-N or Asp/Glu-COO^–^ donor; if the complex has intermediate stability, [VOL]_*n*_-protein adducts can be detected where the binding occurs through the coordination of two or more amino acid side chains in two adjacent equatorial positions. Stable square pyramidal [V^IV^OL_2_] complexes, which only have a weak coordinative axial site, can interact by noncovalent interactions (van der Waals contacts and hydrogen bonds) with accessible groups on the protein surface.^34^ The main limitation of previous studies lay in identifying the specific protein donors involved in metal coordination and the stability of the various adducts detected by ESI-MS. A more detailed molecular characterization can be obtained by multistage MS^*n*^ experiments, as previously shown for various metal complexes.^26,27,35−37^

**Scheme 1:**
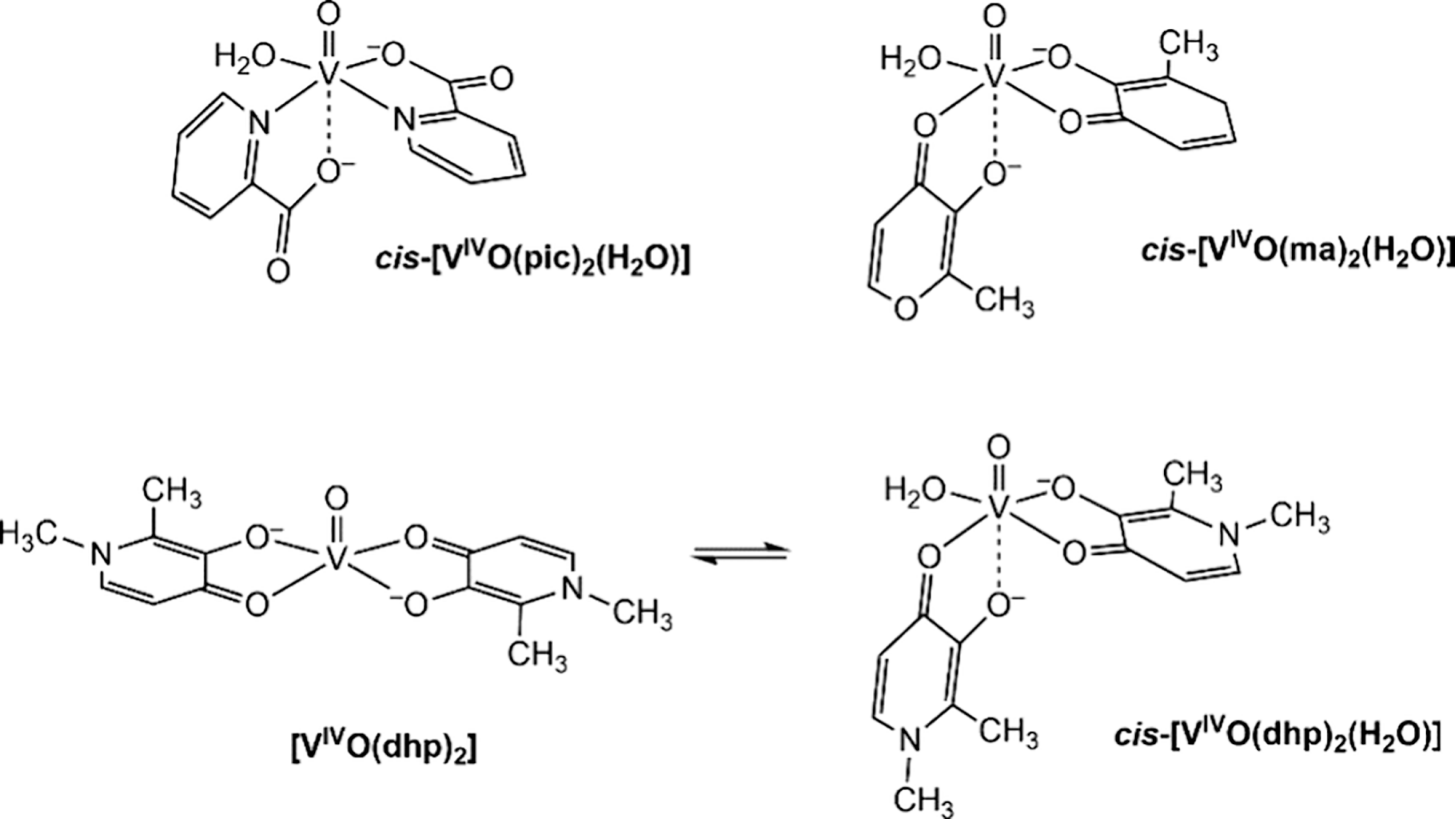
Structures of [V^IV^O(pic)_2_], [V^IV^O(ma)_2_], and [V^IV^O(dhp)_2_] in Aqueous Solution (mM Concentration)

In the current study, we aimed to gain new insights into metal-peptide adduct formation and binding site preferences of coordinated vanadium-peptide adducts using a MS^2^-based approach and apply this technique to kinetically labile first-row transition metal compounds. The stepwise increase of the normalized collision energy enabled insights into the stability of the vanadium-peptide adducts. By combining these findings with EPR measurements and computational methods, we were able to provide important and unique in-depth information on the [V^IV^OL_2_]-biospeciation (L = dhp, ma, pic) of three vanadium compounds (Scheme 1) with the model peptides angiotensin 2 (AT2) and angiotensin 1 (AT1). Our results with model systems suggest that this tandem mass spectrometry approach could be further extended to more relevant and complex target proteins.

## Experimental Section

### Materials

Water was deionized prior to use through a Millipore Milli-Q academic purification system. The chemicals oxidovanadium(IV) sulfate trihydrate ([V^IV^OSO_4_·3H_2_O]), pyridine-2-carboxylic acid (picolinic acid), 3-hydroxy-2-methyl-4*H*-pyran-4-one (maltol), 1,2-dimethyl-3-hydroxy-4(1*H*)-pyridinone (deferiprone), 1-methylimidazole (MeIm), 4-(2-hydroxyethyl)-piperazine-1-ethanesulfonic acid (HEPES), angiotensin I (AT1, H_2_N-Asp-Arg-Val-Tyr-Ile-His-Pro-Phe-His-Leu-OH), and angiotensin II (AT2, H_2_N-Asp-Arg-Val-Tyr-Ile-His-Pro-Phe-OH) were Sigma-Aldrich products of the highest grade available and used as received; acetonitrile (ACN) (LC-MS quality, Sigma-Aldrich) and formic acid (FA) (LC-MS quality, Sigma-Aldrich) were used without further purification. The complexes [V^IV^O(dhp)_2_], [V^IV^O(ma)_2_], and [V^IV^O(pic)_2_(H_2_O)] were synthesized following the procedures established in the literature.^24,38,39^

### EPR

The aqueous solutions for EPR measurements were prepared by dissolving [V^IV^OSO_4_·3H_2_O] and the ligand (L = ma, dhp, and pic) in ultrapure Milli-Q water to get a [V^IV^O]^2+^ concentration of 2.0 × 10^–3^ M and a metal-to-ligand molar ratio of 1/2 or 1/1. HEPES buffer of 0.1 M concentration was added, and the pH was adjusted to the desired value. Stock solutions of angiotensin peptide AT (AT refers to both AT1 and AT2) were prepared in Milli-Q water with a concentration of 2.0 × 10^–3^ M. In the ternary systems, an appropriate amount of AT or MeIm was added to the V–L solutions to obtain a [V^IV^O]^2+^/L/AT molar ratio of 1/2/1 or 1/1/1 and [V^IV^O]^2+^/L/MeIm 1/1/1 or 1/2/4, and a V concentration of 1.0 × 10^–3^ M.

Argon was bubbled through all of the solutions to ensure the absence of oxygen and avoid the oxidation of [V^IV^O]^2+^ ions. For frozen solution EPR measurements, DMSO is usually added to the aqueous samples to get a better resolution of the spectra. However, as the addition of this solvent can influence the equilibria in solution and denature the proteins, HEPES was used for EPR measurements at 120 K, which allowed us to achieve a good resolution of the spectra.

The EPR spectra were recorded at 120 K by using an X-band Bruker EMX spectrometer equipped with an HP 53150A microwave frequency counter. The instrumental parameters were as follows: microwave frequency, 9.40–9.41 GHz; microwave power, 20 mW; time constant, 81.92 ms; modulation frequency, 100 kHz; modulation amplitude, 0.4 mT; and resolution, 4096 points. When the samples were transferred to the EPR tubes, the spectra were immediately measured. Signal averaging was used to increase the signal-to-noise ratio. The recorded spectra were simulated with the software Bruker WINEPR SimFonia (version 1.26 (beta), Bruker Analytik GmbH, 1997).

### DFT

DFT calculations were carried out with Gaussian 09 (revision C.01).^40^ The [V^IV^O] complex geometries and their relative stability were computed at the level of theory B3P86/6-311g(d,p); this method guarantees a good degree of accuracy in the structural optimization of first-row transition metal complexes^41,42^ and, particularly, of vanadium compounds.^43^ Water was simulated within the framework of the SMD model.^44^ For bis-chelated V species, the most stable isomers (Scheme S1) were considered in the DFT calculations, as suggested by previous studies^45,46^ and references therein. Monochelated [V^IV^OL(H_2_O)_*x*_] species were also computed since they are formed in solution by ma and pic ligands under the above-described experimental conditions.

The structures of model peptides H_2_N-His-Pro-Ala-His-NH_2_ and H_2_N-His-Pro-Ala-OH were used to simulate the coordination of AT1 and AT2, respectively. The reactions that were used to calculate the relative stabilities of the ternary species (Δ*G*_aq_) can be found in eqs 1–3 (Section, DFT calculations). Optimized structures of ternary species [V^IV^OL_2_(**His**ProAla)] and [V^IV^OL(**His**ProAla**His**)] are reported in Figures S1 and S2.

The Gibbs energy in aqueous solution (*G*_aq_) for each species can be separated into the electronic plus nuclear repulsion energy (*E*_ele_), the thermal contribution (*G*_therm_), and the solvation energy (Δ*G*_solv_): *G*_aq_ = *E*_ele_ + *G*_therm_ + Δ*G*_solv_. The term RT ln(24.46) was considered to account for the standard state correction from the gas phase to the aqueous solution. The thermal contribution was estimated using the ideal gas model and the calculated harmonic vibrational frequencies to determine the correction due to the zero-point energy and thermal population of the vibrational levels.

For the optimized structures of both binary and ternary species, the ^51^V hyperfine coupling constants (*A*) were calculated using the half-and-half hybrid functional BHandHLYP and the basis set 6-311+g(d), according to the procedures previously published.^47−52^ It must be taken into account that for a [V^IV^O]^2+^ species, *A*_*z*_ is usually negative; however, in the literature, its absolute value is often reported and this formalism was also used in this study. The theoretical background is described in detail in refs (53−55). The percent deviation (PD) of the absolute calculated value, *A*_*z*_, from the absolute experimental value, *A*_*z*exptl_, was obtained as follows: 100 × [(*A*_*z*_ – *A*_*z*exptl_)/*A*_*z*exptl_].

### ESI-MS (Electrospray Ionization Mass Spectrometry)

Aqueous stock solutions (2 mM in Milli-Q H_2_O) of the [V^IV^OL_2_] complexes were prepared under an argon atmosphere and mixed with the respective peptide stock solution (500 μM in Milli-Q H_2_O AT2/AT1) to reach a ratio of 4:1 and a final peptide concentration of 100 μM (pH 6). This incubation mixture was further diluted with ACN/H_2_O containing 0.1% FA to a peptide concentration of 50 μM to yield **1**–**6** (Table 1) for the immediate MS measurement.

**Table 1:** Incubation Mixtures of AT + [V^IV^OL_2_] Complexes **1**–**6**

[V^IV^OL_2_]	AT2 systems	AT1 systems
[V^IV^O(dhp)_2_]	**1** AT2 + VO(dhp)_2_	**2** AT1 + VO(dhp)_2_
[V^IV^O(ma)_2_]	**3** AT2 + VO(ma)_2_	**4** AT1 + VO(ma)_2_
[V^IV^O(pic)_2_]	**5** AT2 + VO(pic)_2_	**6** AT1 + VO(pic)_2_

HR-mass spectra were recorded on an Orbitrap Elite ESI-MS instrument (Thermo Scientific) in positive ion mode. Typically, sample solutions were infused at 5 μL/min and ionized in the HESI (heated electrospray ionization) source with standard conditions (HESI temperature 45 °C, 3–4 kV spray voltage, capillary temperature 275 °C, and sheath gas flow rate at 5 arbitrary units). Precursor ions were typically selected with an *m*/*z* window of 5 Da and were subjected to increasing amounts of normalized collision energy in the HCD cell. Data analysis was performed using the Xcalibur software package (Thermo Scientific) and the Apm^2^s software tool.^56^

## Results and Discussion

**Figure 1:**
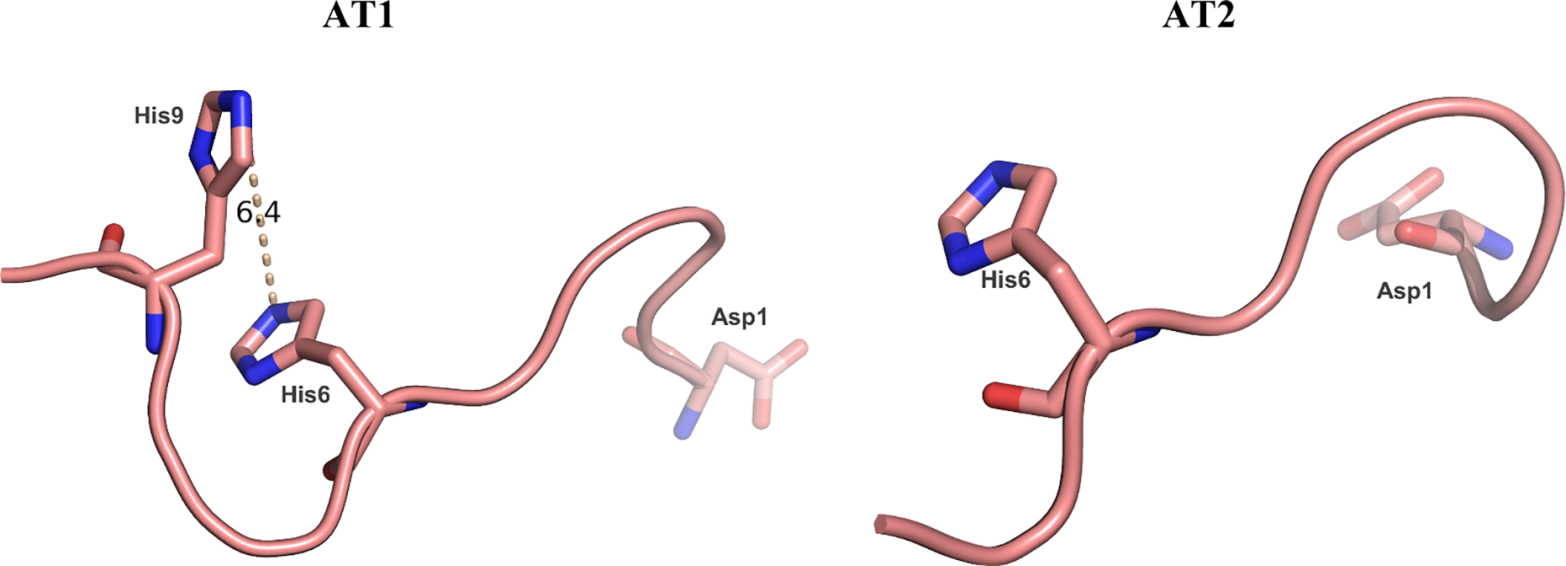
Potential binding sites for vanadium in AT2 and AT1 in the stick presentation. Left: His6, His9 as potential polydentate binding site and Asp1 as monodentate binding site in AT1; Right: His6 and Asp1 as potential monodentate binding sites in AT2.

At physiological pH, the imidazole-*N*^ε^ of histidine residues (*N*_His_^ε^) are expected to be good donor groups for vanadium complexes.^4,37^ This, together with the high affinity toward oxygen donors, such as aspartic acid (Asp), glutamic acid (Glu), and tyrosine (Tyr), has been reported in several preliminary studies on bis-chelated oxidovanadium(IV) complexes.^6,7,10,57−60^ Angiotensin was chosen as a model peptide, as the 10 amino acid sequence of AT1 contains a His6-X-X-His9 motif, while AT2 is truncated by two amino acids and only contains His6. Figure 1 shows the peptide structures of AT2 and AT1 in solution, as derived from the PDB (1N9U and 1N9V). The intramolecular distance between the two *N*_His_^ε^ groups in AT1 is around 6.4 Å, allowing for potential bidentate binding of the complexes. However, it is important to note that the intramolecular distances may vary, given that the peptide structure is inherently flexible. The sequence of AT2 allows for more spatial freedom for potential coordination partners. Asp1 represents another potential binding site on the N-terminus of both peptides for monodentate [VOL_*x*_] (*x* = 0–2) coordination. All potential binding sites within the sequences of AT1 and AT2 are shown as stick presentations in Figure 1.

Considering the different sequence of the two model peptides, the interaction of both AT1 and AT2 with three oxidovanadium(IV) complexes was first investigated by EPR spectroscopy to see if the proposed His donors can be involved in the binding toward the metal. The results were then combined with those obtained by ESI-MS^*n*^ to better describe the molecular interactions.

### EPR and DFT

The behavior of the [V^IV^O(ma)_2_] complex in solution has been studied previously.^61,62^ At mM concentrations and physiological pH, the complex is present in solution as *cis*-[V^IV^O(ma)_2_(H_2_O)] and the equatorial water molecule can be easily replaced by a stronger donor such as N-His or a carboxylate group of Asp or Glu amino acid residues present on a protein surface. The spectra of the bis-chelated *cis*-[V^IV^O(ma)_2_(H_2_O)] (red line in Figure S3) and the ternary complex with *N*-methylimidazole (MeIm), *cis*-[V^IV^O(ma)_2_(MeIm)] (blue line in Figure S3), where MeIm was used as a model for *N*_His_ coordination, were used as references.

The spectrum recorded at pH 6.9 in a ternary system containing [V^IV^O(ma)_2_] and AT1 in a 1/1 ratio resembles that of *cis*-[V^IV^O(ma)_2_(MeIm)], suggesting that the binding of AT1 can occur through the coordination of a His residue. Considering that AT1 contains also a second His residue, which can be involved in the binding, DFT calculations on model complexes *cis*-[V^IV^O(ma)_2_(**His**ProAla)] with one side-chain His residue coordinated to isomers OC-6-32 and OC-6-34 (see Scheme S1), and [V^IV^O(ma)(**His**ProAla**His**)] with two side chain His residues coordinated to [V^IV^O(ma)]^+^, were carried out to obtain the calculated *A*_*z*_ values. The optimized structures of the two coordination modes, designated as model 1 (2 His coordination; HisProAlaHis) and model 2 (1 His coordination; HisProAla), can be found in the Supporting Information (Figure S1). The results summarized in Table S1 show that it is impossible to distinguish which type of binding is predominant in solution. In fact, even if the smaller error is shown by [V^IV^O(ma)(**His**ProAla**His**)], larger errors observed when comparing the experimental and calculated values of MeIm species do not allow to exclude the formation of *cis*-[V^IV^O(ma)_2_(**His**ProAla)].

The results obtained when using a [V^IV^O]^2+^/ma/AT1 at a 1/1/1 ratio are shown in Figure S4. In these experimental conditions, the binding to AT1 should be favored since the amount of the monochelated complex [V^IV^O(ma)(H_2_O)_*x*_] is higher than in the system [V^IV^O]^2+^/ma/AT1 1/2/1 shown in Figure S3. Again, with the spectrum of the species existing at pH 7 in the system [V^IV^O]^2+^/ma/AT1 at a 1/1/1 ratio (blue line in Figure S4), the differences are small in comparison to the spectrum obtained in the system [V^IV^O]^2+^/ma/MeIm at a 1/1/1 ratio (green line in Figure S4). Decreasing the concentration, which should shift the equilibrium toward the monochelated complex, there is no detectable effect on the spectrum (red line in Figure S4).

Therefore, experimental evidence obtained with EPR spectroscopy at different [V^IV^O]^2+^/ma/AT1 ratios indicates that it is impossible to determine whether the formation of mixed complexes with AT1 occurs through the coordination of one or two His residues.

The behavior of the [V^IV^O(pic)_2_(H_2_O)] complex has already been studied in solution; around neutrality, the following equilibrium exists in solution:
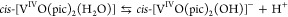


The p*K*_a_ of the equatorially coordinated water molecule is 6.98.^63^ The equilibrium is shifted toward the formation of the ternary species *cis*-[V^IV^O(pic)_2_(MeIm)] when MeIm is present in solution to mimic the side-chain His coordination.^64^

**Figure 2:**
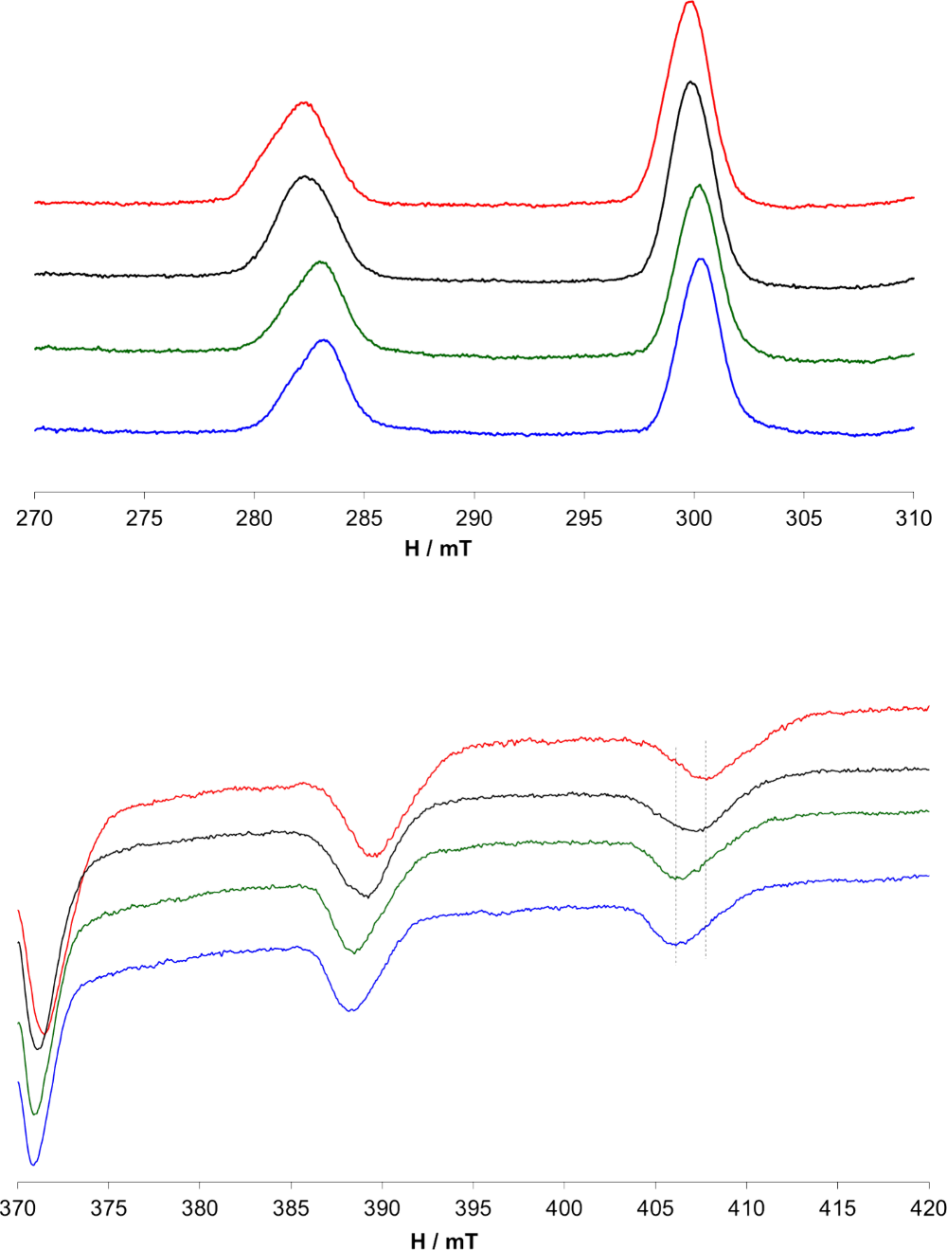
Low-field (top) and high-field (bottom) regions of the anisotropic EPR spectra recorded on frozen solutions containing [V^IV^O]^2+^/pic 1/1 ([V^IV^O]^2+^ 1 mM), red line; [V^IV^O]^2+^/pic/AT1 1/1/1 ([V^IV^O]^2+^ 1 mM), black line; [V^IV^O]^2+^/pic/AT2 1/1/1 ([V^IV^O]^2+^ 1 mM), green line; and [V^IV^O]^2+^/pic/MeIm 1/1/1 ([V^IV^O]^2+^ 1 mM), blue line.

The anisotropic EPR spectra recorded on the ternary system [V^IV^O]^2+^/pic/AT1 with a 1/1/1 ratio are shown in Figure 2. As can be inferred from Table 2, the *A*_*z*_ value of the monohydroxido species, *cis*-[V^IV^O(pic)_2_(OH)]^−^, is very similar to that of the MeIm-coordinated species. The spectra recorded at pH 7.1 with and without AT1 are different from each other and also from the spectrum of the system [V^IV^O]^2+^/pic/MeIm 1/1/1. In the latter conditions, a mixture of *cis*-[V^IV^O(pic)_2_(H_2_O)], *cis*-[V^IV^O(pic)_2_(OH)]^−^, and *cis*-[V^IV^O(pic)_2_(MeIm)] exists in solution.

**Table 2:** Experimental (*A*_*z*exptl_) and DFT-Calculated (*A*_*z*_) Spin Hamiltonian Parameters for V Complexes

	*A*_*z*_^a^	*A*_*z*exptl_^a^	err %^b^
[V^IV^O(pic)_2_(H_2_O)] (OC-6-23)	–163.50^c^	–163.8^c^	–0.2
[V^IV^O(pic)_2_(H_2_O)] (OC-6-24)	–160.94^c^		–1.7
[V^IV^O(pic)_2_(OH)]^−^ (OC-6-23)	–154.19^c^	–160.7^c^	–4.1
[V^IV^O(pic)_2_(OH)]^−^ (OC-6-24)	–154.32^c^		–4.0
[V^IV^O(pic)_2_(N-MeIm)] (OC-6-23, d 211°)	–155.31^c^	–160.0^d^	–2.9
[V^IV^O(pic)_2_(N-MeIm)] (OC-6-24, d 188°)	–155.88^c^		–2.6
[V^IV^O(pic)(**His**ProAla**His**)]	–163.05	–162.3^e^	0.5
[V^IV^O(pic)_2_(**His**ProAla)] (OC-6-24)	–155.16	–160.4^f^	–3.3
[V^IV^O(pic)_2_(**His**ProAla)] (OC-6-23)	–155.47	–160.4^f^	–3.1

a A values reported in 10^–4^ cm^–1^.

b Percent deviation (PD) with respect to the absolute experimental *A*_*z*_ value calculated as 100 × [(|*A*_*z*_| – |*A*_*z*exptl_|)|/|*A*_*z*exptl_|].

c From ref (46). In brackets, the isomer and the dihedral angles between the imidazole ring and the V=O bond are reported.

d This work. Measured in the system [V^IV^O]^2+^/pic/MeIm 1/1/1 ([V^IV^O]^2+^ 1 mM).

e This work. Measured in the system [V^IV^O]^2+^/pic/AT1 1/1/1 ([V^IV^O]^2+^ 1 mM).

f This work. Measured in the system [V^IV^O]^2+^/pic/AT2 1/1/1 ([V^IV^O]^2+^ 1 mM).

To understand whether AT1 coordinates the metal through one or two His, EPR measurements were also carried out with AT2. The spectrum of the ternary system [V^IV^O]^2+^/pic/AT2 with the 1/1/1 ratio, shown in Figure 2, is different from that recorded with AT1, while it is similar to that recorded in the ternary system [V^IV^O]^2+^/pic/MeIm with a 1/1/1 ratio. An explanation for the different behavior of these two peptides is that AT2, having only one His residue coordinates like MeIm, while AT1 having two His residues, is able, at least partially, to replace one pic ligand with formation of a species with [V^IV^O(pic)(N_His_, N_His_)] coordination.

DFT calculations on model complexes [V^IV^O(pic)(**His**ProAla**His**)], with two side-chain His residues coordinated to [V^IV^O(pic)]^+^, and *cis*-[V^IV^O(pic)_2_(**His**ProAla)], with one side-chain His residue coordinated to isomers OC-6-23 and OC-6-24 (see Scheme S1), were carried out to obtain the calculated *A*_*z*_ values. The optimized structures of model complexes considered in the calculations are shown in Figure S2. The results summarized in Table 2 show that the calculated *A*_*z*_ value of [V^IV^O(pic)(**His**ProAla**His**)] is very similar to the experimental value measured in the system with AT1, while for *cis*-[V^IV^O(pic)_2_(**His**ProAla)], the calculated hyperfine splitting constant is similar to that measured in the system with AT2 and the analogous species with MeIm.

Therefore, both EPR spectra and DFT calculations indicate that AT2, having only one His residue, behaves like a monodentate donor like MeIm, or HisProAla, while AT1, with two His residues, gives a spectrum, or a calculated *A*_*z*_ value, different from that obtained with AT2 and also by that obtained in the binary system [V^IV^O]^2+^/pic.

EPR spectra with [V^IV^O(dhp)_2_] were not measured since the complex is more stable at this concentration than those formed by pic and ma, and no relevant evidence of peptide binding by EPR is expected, except for monodentate coordination of His to *cis*-[V^IV^O(dhp)_2_].

*ESI-MS measurements* enable the investigation of interactions between metal complexes and model peptides within the physiologically relevant micromolar range, providing a significant advantage over other methods, such as EPR and XRD experiments.^7^

Previous results^7,10,29,32^ with model proteins (ubiquitin, cytochrome, lysozyme, and myoglobin) showed that [V^IV^O(ma)_2_] formed adducts with the stoichiometry [V^IV^O(ma)]_*n*_-protein, while with [V^IV^O(pic)_2_(H_2_O)] and [V^IV^O(dhp)_2_], both adducts [V^IV^OL]_*n*_–protein and [V^IV^OL_2_]_*n*_–protein were observed. The different behavior was explained considering the stability of the compounds at micromolar concentrations, which favored the hydrolysis of the bis-chelated complex and the formation of [V^IV^OL(H_2_O)_*x*_]. The potentiometric data in the literature^29^ indicate that [V^IV^O(dhp)_2_] and [V^IV^O(pic)_2_(H_2_O)] are more stable than [V^IV^O(ma)_2_] and partially survive in solution even at micromolar concentrations; they can bind to proteins as 1:2 species, while [V^IV^O(ma)_2_] undergoes hydrolysis and only the 1:1 moiety [V^IV^O(ma)] interacts with the protein.

**Figure 3:**
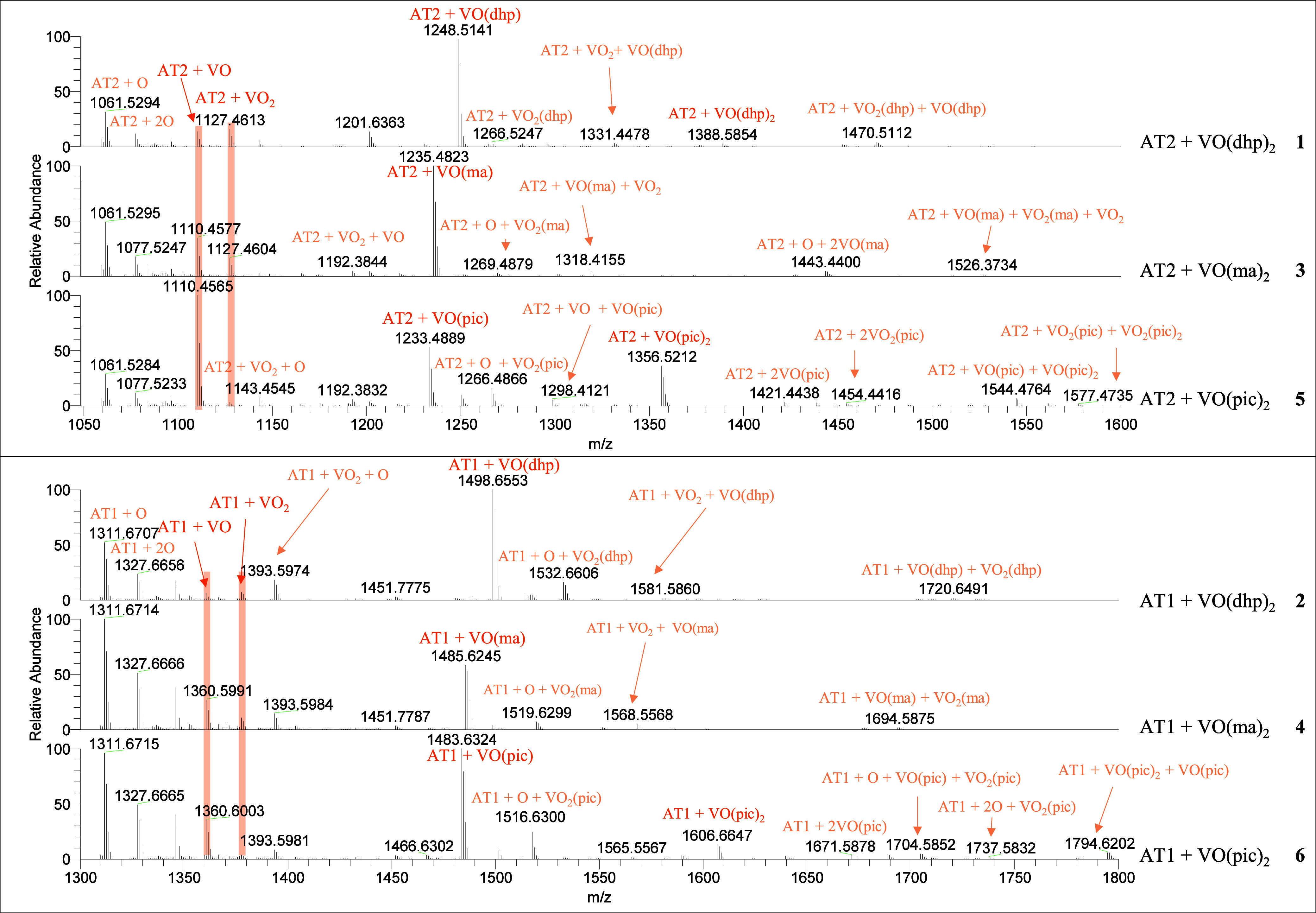
Zoom into the relevant range of the deconvoluted mass spectra for AT2 and AT1 with [V^IV^O(dhp)_2_] (**1** + **2**), [V^IV^O(ma)_2_] (**3** + **4**), and [V^IV^O(pic)_2_] (**5** + **6**).

To examine the adduct speciation of [V^IV^O(dhp)_2_], [V^IV^O(ma)_2_], and [V^IV^O(pic)_2_], full-MS spectra were recorded immediately after incubation with AT2 and AT1 under an argon atmosphere. The analysis was repeated at various time points, but no significant differences were observed over time. To prevent any interference from size and charge differences between AT2 and AT1, charge-independent mass spectra were calculated. The corresponding deconvoluted spectra are shown in Figure 3, with the abundances of the major adduct species relative to the pure peptides listed in Table 3. Please refer to Tables S2–S7 in the Supporting Information for comprehensive peak lists of all identified species along with their experimental ppm error. Despite working under an argon atmosphere, inevitable vanadium oxidation occurred during experimental ESI-MS conditions, as previously observed.^10,29,30,57,65,66^ Please note that most of the found adduct peaks were overlapping signals of V^IV^ and V^V^ species (see Figures S9–S14) and were not distinguished due to the complexity of the spectra. The exact oxidation state will be discussed in detail in the MS^2^ part.

**Table 3:** Relative Abundances in % of Major Adduct Species in Deconvoluted Mass Spectra Relative to the Pure Peptide Peaks (Base Peak, AT2 *m*/*z* = 1045; AT1 *m*/*z* = 1295)

adduct ion	**AT2/[V**^**IV**^**O(dhp)**_**2**_**]**	**AT1/[V**^**IV**^**O(dhp)**_**2**_**]**	**AT2/[V**^**IV**^**O(ma)**_**2**_**]**	**AT1/[V**^**IV**^**O(ma)**_**2**_**]**	**AT2/[V**^**IV**^**O(pic)**_**2**_**]**	**AT1/[V**^**IV**^**O(pic)**_**2**_**]**
AT	100	100	100	100	100	100
[AT + VOL]	**4.35**	**4.53**	**4.49**	**2.02**	2.82	**3.67**
[AT + VOL_2_]	0.11	0.00	0.02	0.00	1.92	0.48
[AT + VO]	0.60	0.34	1.55	0.92	**5.32**	1.29
[AT + VO_2_]	0.70	0.31	0.72	0.37	0.16	0.09

In the spectra recorded with both AT1 and AT2 (Figure 3), [AT + VOL] peaks were observed to be the dominant adduct species for most systems. Thus, the coordination of the peptides appears to replace one of the ligands for the V complexes during adduct formation. However, as shown in Table 3, adducts with stoichiometry [AT + VOL_2_] were observed as low-abundance species mainly with AT2. In the latter case, coordination through a side-chain amino acid residue to the *cis*-[VOL_2_] fragment is possible. In addition, various adducts containing [VO]/[VO_2_] moieties were identified, and both peptides seem to be able to bind up to two/three metal fragments simultaneously. In particular, various multimetalated adducts, such as [AT + *m*VO_*n*_ + *p*VO_*n*_L+ *q*VOL_2_] (*m* = 0–1, *n* = 1–2; *p* = 0–2; *q* = 0–2), were detected in the incubation mixtures even if they show low relative abundance. Comparing the three [VO]^2+^ complexes, multimetalated species are more abundant in the systems containing the pic complex (Figure 3, spectra **5** and **6**).

Some general conclusions can be drawn from the speciation of the vanadium compounds and the different coordination behavior of the two peptides under examination. The behavior of the three vanadium compounds with AT2 resembles what was previously observed with model proteins, and which can be explained considering the different distributions.^29^ Low concentrations favor hydrolysis of the bis-chelated complexes, which is more pronounced in the case of ma, the weaker ligand, compared to dhp and pic.^29^ Specifically, with ma no adducts with the bis-chelated [VOL_2_] were observed, while for dhp and pic, both [VOL]/[VOL_2_] moieties were found bound to the peptide. This is in agreement with previous data.^7,29^

However, when the more stable dhp/pic complexes were incubated with AT1, the relative abundance of [AT1 + VOL_2_] signals was lower compared to that of the corresponding [AT2 + VOL_2_] signals (Figure 3 and Table 3). Especially for the pic system, [AT1 + VO(pic)_2_] was only present with 0.48% relative abundance while [AT2 + VO(pic)_2_] reached 1.92% relative abundance. In contrast, it can be noticed that [AT2 + VO(pic)] abundance is lower than the corresponding signal with AT1 (relative abundance 2.82 vs 3.67, Table 3 and Figure 3). For dhp, the different behavior toward the two peptides could be observed from the lack of the signal [AT1 + VO(dhp)_2_].

These observations support the hypothesis mentioned in the EPR section that AT1 and AT2 exhibit different coordination behaviors toward the metal complexes. The preference to bind the [VOL] fragment in the case of AT1 can be explained with the presence of two His residues, which can both coordinate to the metal ion in a bidentate fashion replacing one ligand. The binding with AT2 can occur through the monodentate coordination of one His to the *cis*-[VOL_2_] moiety replacing a water molecule coordinated in the equatorial position, or with hydrolytic species [VO_*x*_]/[VOL] involving other amino acid residues in the binding to the metal center.

#### Tandem-MS Fragmentation

Follow-up MS^2^ fragmentation experiments on an isolated ion of interest have been proven to be a powerful method for characterizing binding site preferences within model peptides in several previous studies on different metal complexes.^27,36,37^ Here, this technique has been exploited for the first time to investigate the behavior of vanadium complexes, enabling an in-depth study of binding preferences and hinting at metal-peptide adduct stability. The MS/MS spectra of the most significant metal adducts were recorded by using higher energy collisional dissociation (HCD) with a stepwise increase in normalized collision energy (NCE). In all systems, peptide fragmentation was observed above NCE 30, like for the unmodified peptide (see Figure S5). According to the mobile-proton model,^67^ the peptide bond cleavage resulted in [b]^+^ and [y″]^+^ fragment series, which was detected in the positive ion mode. Eventually, the loss of carbon monoxide resulted in highly abundant a-fragments, while the abundancy of C-terminal z-fragments after deamination was less pronounced. Coordinative bonds to the charged vanadium complex led to the replacement of protons as charge carriers, which was tracked in the respective sum formulas of the fragments. However, the exact molecular processes occurring in the fragmentation of metalated peptide precursor ions remain elusive. In Figure S6, the peptide fragmentation nomenclature established by Roepstorff and Fohlman^68^ in 1984 and the proposed procedure to monitor the formal oxidation state of vanadium is illustrated.

**Figure 4:**
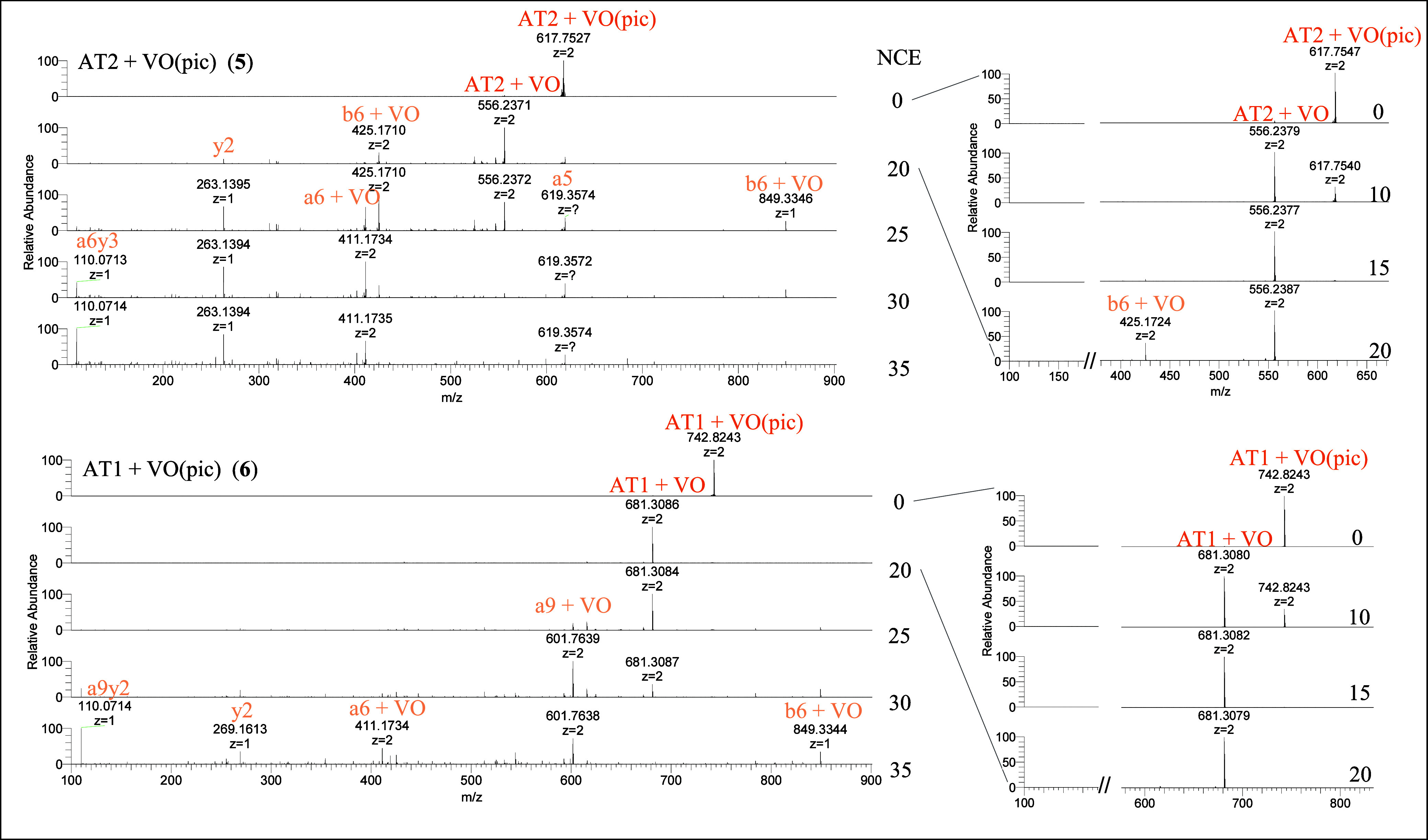
Fragmentation behavior of [AT2 + VO(pic)] (top) and [AT1 + VO(pic)] (bottom) precursor ions at NCE 0–35.

In general, loss of the ma and pic ligands was observed at low collision energy (NCE 10), leading to [AT + VO] ions, which further dissociated at higher NCE values, enabling binding site identification of the [VO] moiety (see Figure 4 and Figure S8). The [AT + VO(dhp)] precursor ions instead were observed to be stable up to higher energies (NCE = 20) (see Figure S7).

In order to better understand the different binding of AT1 and AT2 toward metal species, the fragmentation pattern at increasing NCE values was analyzed in detail and compared for all [AT + VOL] parent ions. The fragmentation of [AT1 + VO(pic)] shows a remarkable stable [a9 + VO] (D-R-V-Y-I-**H-**P-F-**H**) ion that remains as the base peak up to NCE 30 with little other fragments observed at this energy (Figure 4, bottom). AT1 alone (see Figure S5) and the corresponding [AT2 + VO(pic)] precursor ion (Figure 4, top) shows significantly more fragmentation at NCE 30 and below. This points to a gas phase structure of the [a9 + VO] ion that is stabilized by the presence of VO, presumably by bidentate binding of both His residues. While the same [a9 + VO] ion emerges with high abundance in the spectra of AT1 with [VO(ma)] and [VO(dhp)], more other fragments are observed at NCE 30, indicating that the original ligand may influence the binding geometry and stability of the [VO] moiety with the peptide.

**Figure 5:**
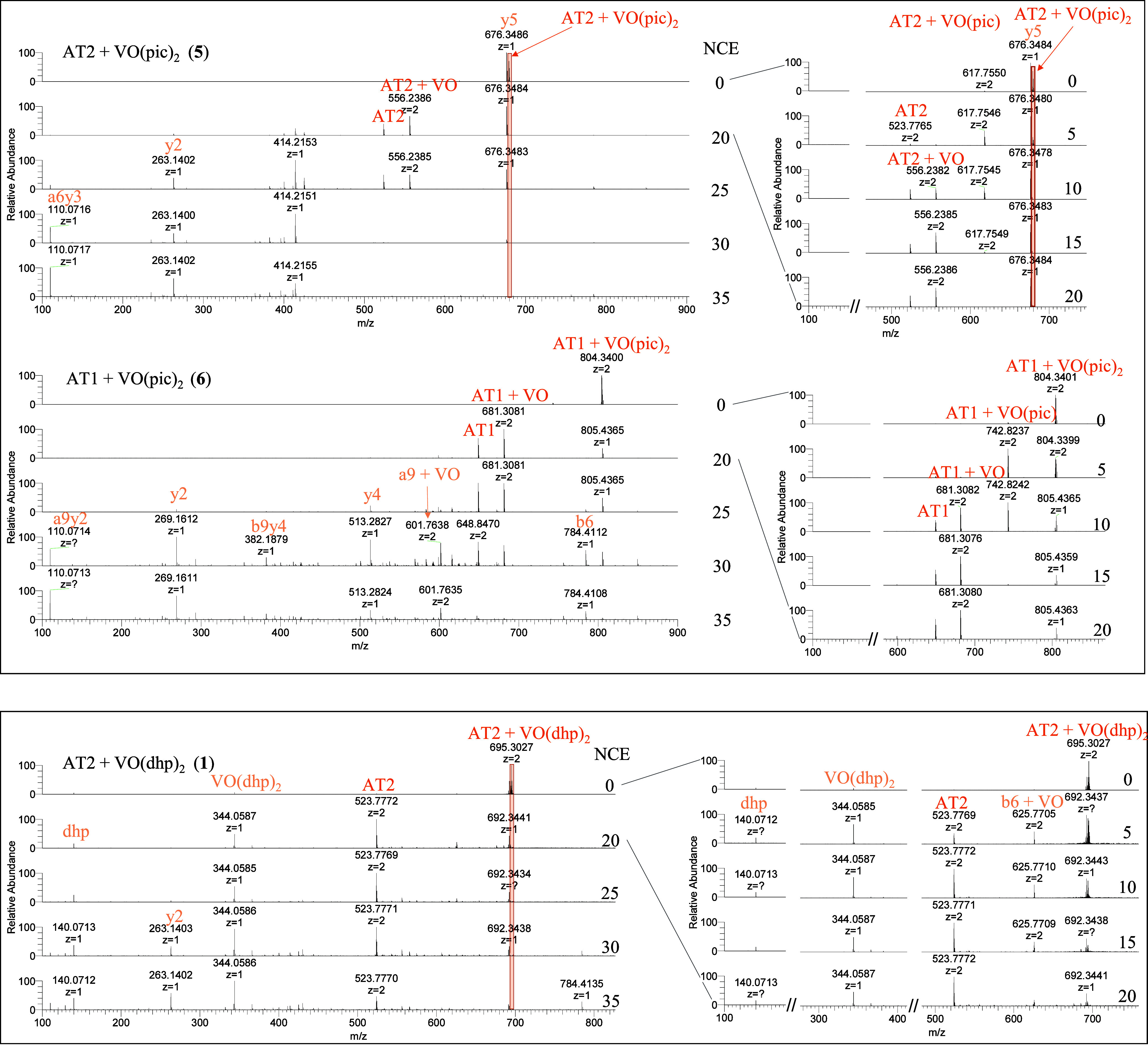
Fragmentation behavior of [AT2 + VO(pic)_2_], [AT1 + VO(pic)_2_] (top), and [AT2 + VO(dhp)_2_] (bottom) precursor ions at NCE 0–35.

Another aspect is the determination of the exact coordination environment in [AT + VOL_2_] species since the complexes can assume different geometries in solution and the binding to the AT peptide can occur through the monodentate coordination of an amino acid side chain or by noncovalent interactions. In previous studies, these possibilities were investigated comparing the MS data with other experimental (EPR spectroscopy) and computational techniques (DFT and docking calculations) for different V compounds and proteins.^28^ In the current study, tandem mass spectra were used as a new tool to better understand the stability and mode of binding in the [AT + VOL_2_] adducts. Figure 5 shows the HCD spectra of [AT2 + VO(pic)_2_], [AT1 + VO(pic)_2_], and [AT2 + VO(dhp)_2_]. The relative abundances of the [AT + VOL_2_] precursor ions in the other three systems were found to be not sufficient to isolate and fragment (see Table 3 and Figure 3). A notable case is the comparison between pic and dhp complexes. Comparing the MS/MS fragmentation at increasing NCE in the systems with AT2, it can be noticed that for dhp, the loss of the entire complex [V^IV^O(dhp)_2_] occurs at low energies and the signal of the free peptide appears together with some hydrolytic species such as dhp (Figure 5). On the other hand, with [AT2 + VO(pic)_2_], the loss of the two pic ligands is observed with [AT2 + VO] being the major metalated species. A similar behavior was observed for [AT1 + VO(pic)_2_]. The absence of the free [VO(pic)_2_] ion (311 *m*/*z*) can be ascribed to the higher stability of this peptide adduct compared with the one with dhp. This is in agreement with the different speciation of the two complexes since dhp forms both *cis*-octahedral *cis*-[V^IV^O(dhp)_2_(H_2_O)] and square pyramidal [V^IV^O(dhp)_2_]. AT can bind the square pyramidal complex [V^IV^O(dhp)_2_] with secondary interactions leading to less stable adducts in comparison to those formed by the *cis*-[V^IV^O(dhp)_2_] and *cis*-[V^IV^O(pic)_2_] moieties, which are stabilized by a covalent bond.

The determination of the specific metal binding site and vanadium oxidation state was done by analyzing the MS/MS spectra in the NCE range 20–35 where peptide fragmentation occurs. The precursor ions were found to form a ligand-dependent equilibrium between [AT + V^IV^OL + H]^2+^ and [AT + V^V^OL]^2+^ prior to fragmentation (Figures S9–S14). Complexes with dhp ligands appear to be most prone to oxidation in this experimental setup, followed by maltol-chelated complexes. Pic complexes in mixtures **5** and **6** were observed to be most resistant toward oxidation. [AT + VOL]^2+^ precursor ions, which represent the predominant vanadium coordination species, were isolated and fragmented for all of the systems.

**Figure 6:**
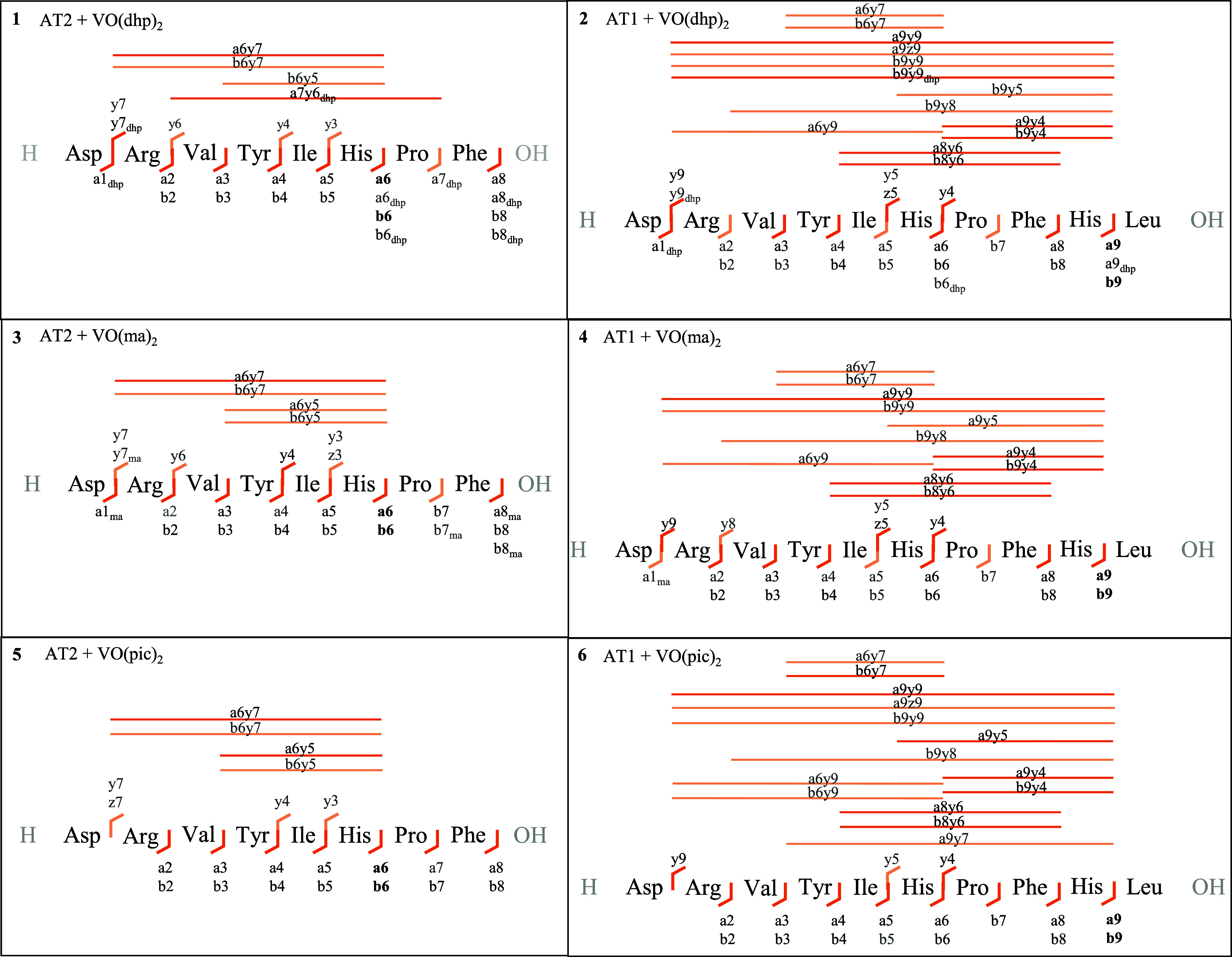
Fragmentation maps of metalated [VOL_*x*_] (*x* = 0–1) peptide fragments in the MS^2^ spectra of [AT + VOL] precursor ions for NCE 20–35 in **1**–**6**; black font: fragments above 1.0% relative abundance; gray font: fragments below 1.0% relative abundance, bold font: most abundant fragments.

Figure 6 shows fragmentation maps that display the identified metalated fragments over an average of NCE 20–35. The relative abundances of the major metalated fragments are listed in Table 4. For more detailed information about all identified metalated fragments together with the respective vanadium oxidation state, please refer to Tables S8–S13 and Figure S6 for the nomenclature of fragments.

**Table 4:** Relative Abundances in % of Major Adduct Species in Fragment Spectra for Systems **1**–**6**

		AT2			AT1		
species	V^i^	**1**	**3**	**5**	**2**	**4**	**6**
[a6 + VO - H]^2+^	V^IV^	**18.56**	**27.84**	**93.57**	1.87	3.71	7.25
[b6 + VO - H]^2+^	V^IV^	**10.51**	**29.12**	**57.21**	3.13	5.19	6.90
[b6 + VO - 2H]^+^	V^IV^	5.04	7.22	21.41	4.65	7.39	12.26
[a9 + VO - H]^2+^	V^IV^				**14.67**	**25.01**	**31.93**
[b9 + VO - H]^2+^	V^IV^				**7.62**	**14.98**	**18.34**
[y4″ + VO - 2H]^+^	V^IV^	0.90	0.40	0.91	1.83	2.42	3.07
[y4″ + VO - H]^+^	V^III^	0.86	1.33				
[y3″ + VO - 2H]^+^	V^IV^	0.49	0.46	0.25			
[z3 + VO - H]^+^	V^III^		0.72				
[y5″ + VO - 2H]^+^	V^IV^				0.76	0.76	0.69
[z5 + VO - H]^+^	V^III^				4.76	2.06	
[a5 + VO - 2H]^+^	V^IV^	2.62	1.21	13.97	0.23	0.34	1.03
[b5 + VO - 2H]^+^	V^IV^	1.58	1.60	8.29	0.23	0.41	0.81

A notable characteristic of all fragmentation experiments (Figure 6 and Table 4) is the presence of highly abundant metalated a6/b6 fragments (**D**-R-V-Y-I-**H**) in AT2 and metalated a9/b9 fragments (**D**-R-V-Y-I-**H**-P-F-**H**) in AT1, which strongly indicate the expected coordination of His in both peptides. As shown in Table 4, the relative abundance of metalated a9/b9 fragments in AT1 is significantly higher than that for a6/b6, suggesting that His9 is involved in the metal coordination.

In AT1 a monodentate coordination of His9 is indicated by metalated C-terminal y4 (P-F-**H**-L) adducts (Figure 6, Table 4; **2**, **4**, and **6**). For AT2, the metalated y4 (I-**H**-P-F) adduct ions further point to His6 coordination. Several metalated small N-terminal a_*n*_/b_*n*_ (*n* = 1–5; **D**-R-V-Y-I) adducts indicate the formation of a coordination bond between a vanadium complex and Asp1 as a second (less occupied) binding site, as suggested in Figure 1. Z-fragmentation under HCD conditions is less pronounced than cleavage of the peptide chain, resulting in y-fragments. However, some metalated z5 (**H**-P-F-**H**-L) adducts in AT1 (Figure 6, **2**+**4**) and a metalated z3 (**H**–P-F) fragment in AT2 (Figure 6, **3**) could be observed. As can be inferred from Table 4, the vanadium moiety is here reduced to [V^III^O] suggesting metalated His6 to support z-fragmentation and reduction of the metal center. Metalated [z5 + V^III^O] (Table 4, **2**+**4**) and [y5 + V^IV^O] (Table 4, **2**, **4**, and **6**) in AT1 further point to a bidentate coordination of both His residues in AT1.

### DFT Stability Calculations

To confirm the stability of the adducts with one or two side-chain His residues bound to [VOL]/[VOL_2_], DFT calculation has been performed considering the formation of mixed complexes [V^IV^OL(**His**ProAla**His**)] and [V^IV^OL_2_(**His**ProAla)]. The following equilibria were considered:
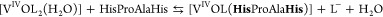
1
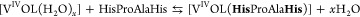
2
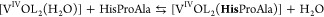
3

Table 5 presents the Gibbs free energy in aqueous solution (Δ*G*_aq_), obtained by DFT calculations for the pic and ma complex coordination to the peptide models. Detailed structures of the two coordination modes, designated as model 1 (2 His coordination; **His**ProAla**His**) and model 2 (1 His coordination; **His**ProAla), can be found in the supporting text (Figures S1 and S2). For the picolinate and maltolate complex, the coordination of AT1 through two His residues was not favored starting from the bis-chelated [V^IV^O(pic)_2_(H_2_O)] and [V^IV^O(ma)_2_(H_2_O)] while it was when starting from the monochelated V species (positive Δ*G*_aq_ values for eq 1 and negative for eq 2). The binding of one His residue (model 2) is less favored, giving slightly negative or positive Δ*G*_aq_ values depending on the isomer (eq 3).

**Table 5:** Δ*G*_aq_ values obtained by DFT calculations for pic- and ma- complexes with peptide coordination models 1 and 2

		Δ*G*_aq_(kcal/mol)	
species	isomer	**His**ProAla**His** (model 1)	**His**ProAla (model 2)
[V^IV^O(pic)_2_(H_2_O)]	OC-6-24	5.18 (eq 1)	1.93 (eq 3)
	OC-6-23	4.55 (eq 1)	–0.99 (eq 3)
[V^IV^O(pic)(H_2_O)_2_]		–10.02 (eq 2)	
[V^IV^O(ma)_2_(H_2_O)]	OC-6-32	9.18 (eq 1)	2.69 (eq 3)
	OC-6-34	7.13 (eq 1)	–0.49 (eq 3)
[V^IV^O(ma)(H_2_O)_2_]		–8.77 (eq 2)	
[V^IV^O(ma)(H_2_O)_3_]		–11.11 (eq 2)	

The experimental results confirm that there is an equilibrium between mono- and bis-chelated species in solution and the predominant species in the MS experimental conditions should be [VOL(H_2_O)_*x*_] (low pH and low total concentrations), which, upon interaction with AT1, forms the species with [V^IV^OL(N_His_, N_His_)] coordination. The optimized structures of [V^IV^OL(**His**ProAla**His**)] show that the N_His_ – N_His_ distance decreases from 6.4 Å in the free peptide to 2.927 (ma) and 2.940 Å (pic). In the same structures, the binding distance V–N_His_ is 2.083 and 2.089 for the maltol complex and 2.070 and 2.092 for the picolinate with N_His_–V–N_His_ angles 89.11° (ma) and 89.87 (pic), confirming that the peptide structure is flexible enough allowing the simultaneous coordination of the two side-chain His residues to the same metal center.

## Conclusions

Understanding the molecular interactions of potential orally applicable antidiabetic drugs based on vanadium with biomolecules is the prerequisite to further vanadium drug development. Screening methods and analytical tools need to be adopted and fine-tuned for specific questions arising from the labile nature of vanadium compounds.

In this study, we combined EPR spectroscopy, theoretical calculations at the DFT level, and ESI-MS/MS to analyze the interactions of three oxidovanadium(IV) compounds with the model peptides AT1 and AT2, which differ in the presence of one or two His residues as potential binding partners.

EPR spectroscopy is a powerful tool to characterize the interaction of [V^IV^O]^2+^ complexes with biorelevant molecules, allowing the determination of geometry and identification of donor groups in the first coordination sphere of the metal ion. However, some limitations due to the complexity of the spectra and interpretation of *A* values can appear, and complementary techniques are often required for comprehensive characterization. In our experiments, EPR spectroscopy did not show clear results for all systems: with [V^IV^O(ma)_2_], it was not possible to determine the formation of mixed complexes with the AT1 peptide. For [V^IV^O(pic)_2_], both EPR and DFT calculations indicated that AT2 interacts as a monodentate His donor, which replaces the water molecule in the equatorial plane, and AT1 resulted in a spectrum and a calculated *A*_*z*_ value pointing toward bidentate binding forming [V^IV^O(pic)(N_His_,N_His_)] ternary species.

ESI-MS/MS analysis was employed to investigate the speciation of a kinetically labile first-row transition metal with medicinal applications in the presence of model peptides. This approach enabled insights into the binding sites of vanadium and showed that the [VO] moiety is tightly bound to the peptide. Our findings indicate that the nature of the coordinated ligand, i.e., ma, dhp, or pic, plays a pivotal role in altering biospeciation and influences the redox behavior of vanadium, which in turn affects the coordination sphere of vanadium within biomolecule adducts.

ESI-MS spectra confirmed that the low concentrations used for MS favor hydrolysis of the bis-chelated complexes, which is more pronounced in the case of weaker ligands, such as ma, where mainly adducts with [VOL] fragments were observed. With stronger ligands, dhp and pic, both [VOL]/[VOL_2_] moieties were found to be bound to the peptides. HCD fragmentation experiments showed the loss of ma and pic ligands at low NCE values, leading to [AT + VO] ions, demonstrating that the vanadium–AT bond is stronger than those with the coordinated ligands ma and pic. The [V^IV^O(dhp)_2_] complex dissociated from the peptide at low NCE values, resulting in a high abundant free peptide peak, whereas [V^IV^O(pic)_2_] resulted in a tightly bound [VO] moiety, which remained attached at NCE 30 after loss of the two pic ligands. Also, the presence of one or two His residues in AT2 and AT1, respectively, resulted in different outcomes. ESI-MS/MS confirmed that AT2 can act as a monodentate binding partner with higher abundant [AT + VOL_2_] adducts, and AT1 can form bidentate interactions leading to more [AT + VOL] adducts. His residues were confirmed as a primary binding partner in all HCD experiments; however, N-terminal side-chain Asp1 binding was observed as well. The presence of a highly abundant [a9 + VO] ion pointed to a gas phase structure stabilized by the [VO] moiety for AT1 by the coordination with two side-chain His residues (His6, His9), while fragmentation of AT2 adducts produced mainly [a6/b6+VO] fragments, suggesting the coordination of His6. Theoretical calculations at the DFT level on model systems that simulate the monodentate coordination of His6 (AT2 model) or bidentate coordination of His6-XX-His9 (AT1 model) confirmed that the binding to [VOL]/[VOL_2_] is thermodynamically favored.

The objective of this study was to enhance the fundamental understanding of biospeciation, redox behavior, and complex geometries of vanadium compounds at the molecular level with model peptides. These investigations enable the application of ESI-MS/MS to more sophisticated systems of vanadium compounds and potential targets, which will guide further vanadium drug development.

## Supporting Information Available

DFT calculations, EPR spectra, HCD fragmentation mass spectra, and tables of identified ions (PDF)
