# Supplementary material for: In-Depth Mass Spectrometry Study of Vanadium(IV) Complexes with Model Peptides
Source: Inorg Chem. 2024 Sep 12;63(38):17785–96. doi: 10.1021/acs.inorgchem.4c02683 (PMC11423397; doi:10.1021/acs.inorgchem.4c02683)
Supplement: Supplementary file 1 — ic4c02683_si_001.pdf [file ic4c02683_si_001.pdf]

Supporting information

In-Depth Mass Spectrometry Study of Vanadium(IV) Complexes with Model Peptides

Kira Küssner,[1] Valeria Ugone,[2]* Daniele Sanna,[2] Monika Cziferszky[1]*

[1] Institute for Pharmacy, Pharmaceutical Chemistry, Department of Chemistry and Pharmacy, University of Innsbruck, Innrain 80/82, A-6020 Innsbruck, Austria

[2]Consiglio Nazionale delle Ricerche, Istituto di Chimica Biomolecolare, Traversa La Crucca 3, 07100 Sassari, Italy

valeria.ugone@cnr.it, monika.cziferszky@uibk.ac.at

Content.

Scheme S1. Most stable isomers of bis-chelated V-species for DFT-calculation**.**

Figure S1. DFT calculation model for one and two His coordination with ma-complex.

Figure S2. DFT calculation model for one and two His coordination with pic-complex.

Figure S3-S4. EPR spectra of $[V^{IV}O]^{2+}$/L/AT systems.

Table S1. Experimental and DFT calculated spin Hamiltonian parameters for V complexes.

Tables S2-7. Identified species in deconvoluted MS^1 spectra for **1-6**.

Figure S5. HCD fragmentation spectra of AT1 and AT2

Figure S6. HCD-Fragmentation pattern of peptides in MS^2; Schematic examples of fragments and the suggested procedure to monitor the formal oxidation state of vanadium.

Figure S7-S8. HCD fragmentation spectra of [AT + VOL] for dhp and ma.

Figures S9-14. Isotopic distribution of the precursor ion [AT + VOL] for **1 – 6** at NCE 0 in the experimental and simulated spectrum to identify vanadium oxidation ratio prior to fragmentation

Tables S8-S13. Identified metalated fragments after fragmentation of $[AT + VOL]^{2+}$ precursor ions in **1 – 6** (NCE 20-35 average), along with the respective vanadium oxidation states.

References.

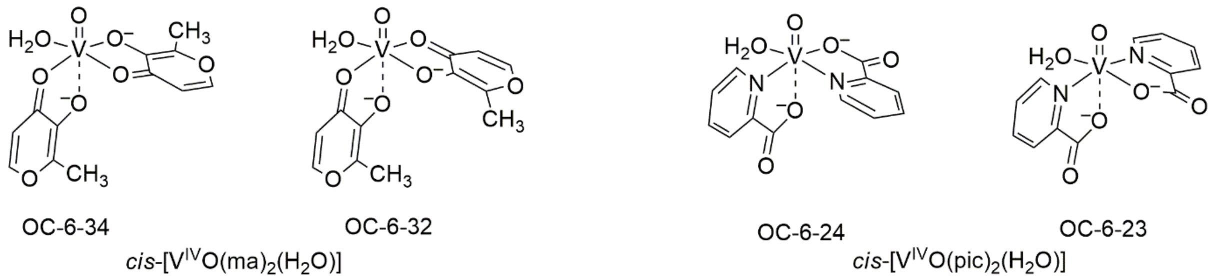

Scheme S1. Most stable isomers of bis-chelated V-species considered for DFT calculations.[1]

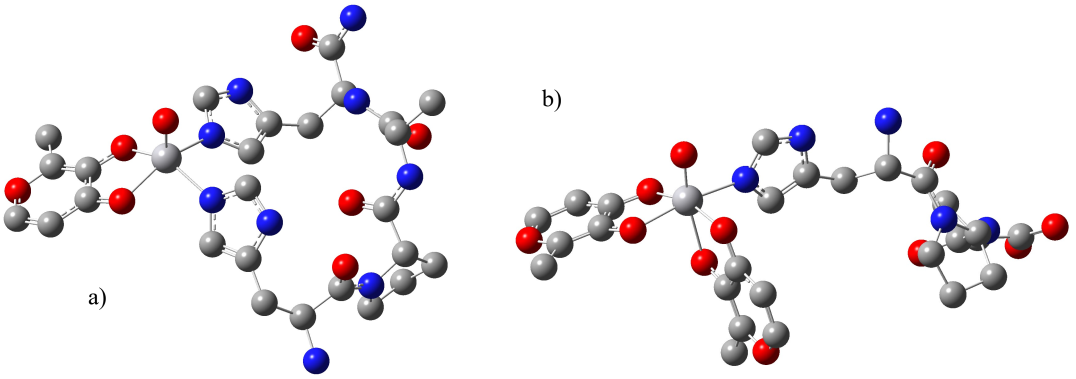

Figure S1: DFT optimized structures of ma-complex with model 1 (2 His coordination) and model 2 (1 His coordination); a) [$V^{IV}O$(ma)(**His**ProAla**His**)] (model 1); b) [$V^{IV}O(ma)_2$(**His**ProAla)] (model 2).

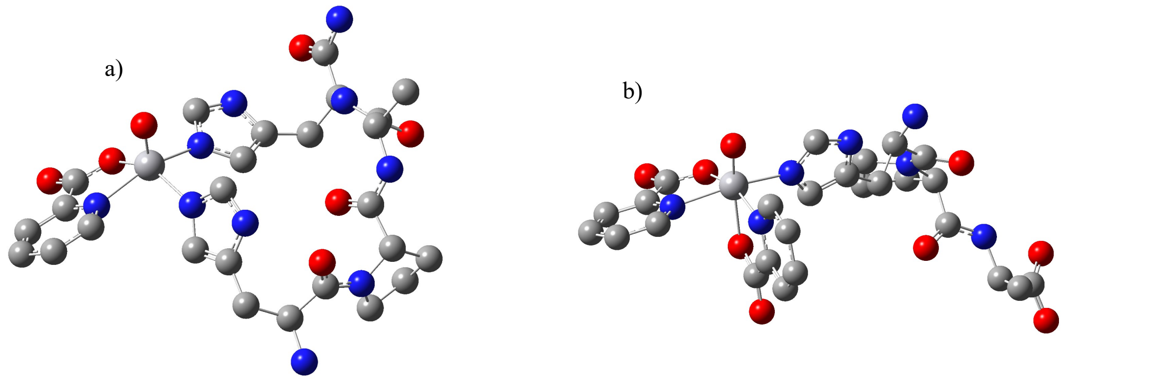

Figure S2: DFT optimized structures of pic-complex with model 1 (2 His coordination) and model 2 (1 His coordination); a) [$V^{IV}O$(pic)(**His**ProAla**His**)] (model 1); b) [$V^{IV}O(pic)_2$(**His**ProAla)] (model 2).

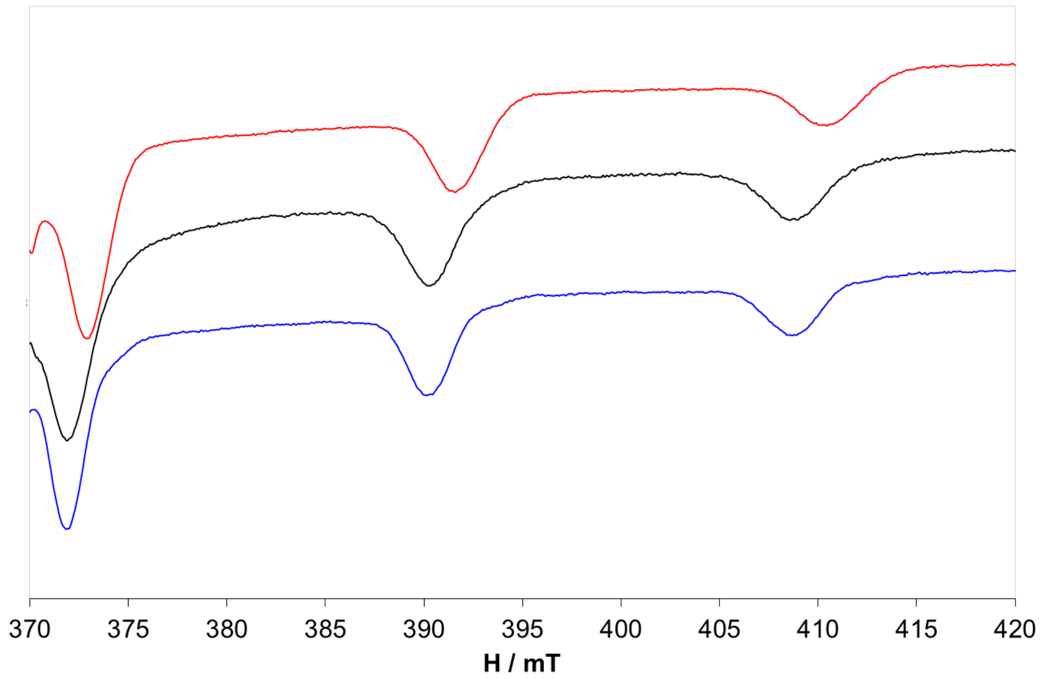

Figure S3. High field region of the anisotropic EPR spectra recorded on frozen solutions containing: $[V^{IV}O]^{2+}$/ma 1/2 ($[V^{IV}O]^{2+}$ 1 mM), red line; $[V^{IV}O]^{2+}$/ma/AT1 1/2/1 ($V^{IV}O^{2+}$ 1 mM), black line; $[V^{IV}O]^{2+}$/ma/MeIm 1/2/1 ($V^{IV}O^{2+}$ 1 mM), blue line.

Table S1. Experimental ($A_{z\ exptl}$) and DFT calculated (A_z) spin Hamiltonian parameters for V complexes.

	A_z	$A_{z\ exptl}$ [a]	err % [b]
$[V^{IV}O(ma)_2(H_2O)]$ (OC-6-32)	-168.61 [c]	-168.9 [c]	-0.2
$[V^{IV}O(ma)_2(H_2O)]$ (OC-6-34)	-168.63 [c]		-0.2
$[V^{IV}O(ma)_2$(N-MeIm)] (OC-6-32, d 3°)	-161.66 [c]	-165.1 [d]	-2.1
$[V^{IV}O(ma)_2$(N-MeIm)] (OC-6-32, d 186°)	-162.11 [c]		-1.8
$[V^{IV}O(ma)_2$(N-MeIm)] (OC-6-34, d 9°)	-162.17 [c]		-1.8
$[V^{IV}O(ma)_2$(N-MeIm)] (OC-6-34, d 198°)	-162.18 [c]		-1.8
$[V^{IV}O$(ma)(**His**ProAla**His**)]	-163.73	-164.9 [e]	-0.7
$[V^{IV}O(ma)_2$(**His**ProAla)] (OC-6-32)	-159.92	-164.9 [e]	-3.0
$[V^{IV}O(ma)_2$(**His**ProAla)] (OC-6-34)	-160.42	-164.9 [e]	-2.7

[a] A values reported in 10^{-4} cm^{-1}. [b] Percent deviation (PD) with respect to the absolute experimental A_z value calculated as: 100 × $[(|A_z| - |A_{z\ exptl}|)/|A_{z\ exptl}|]$. [c] From ref.[2] In brackets the isomer and the dihedral angles between the imidazole ring and the V=O bond are reported. [d] This work. Measured in the system $[V^{IV}O]^{2+}$/ma/MeIm 1/2/1 ($[V^{IV}O]^{2+}$ 1 mM). [e] This work. Measured in the system $[V^{IV}O]^{2+}$/ma/AT1 1/2/1 ($[V^{IV}O]^{2+}$ 1 mM).

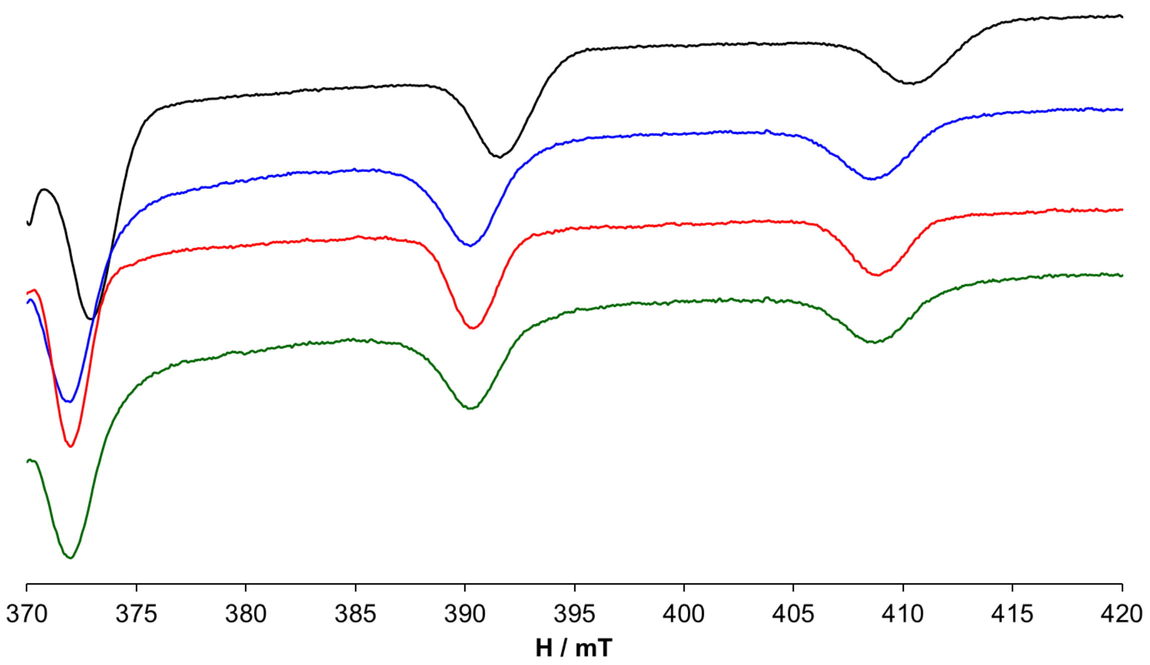

Figure S4. High field region of the anisotropic EPR spectra recorded on frozen solution containing: $[V^{IV}O]^{2+}$/ma 1/2 ($[V^{IV}O]^{2+}$ 1 mM), black line; $[V^{IV}O]^{2+}$/ma/AT1 1/1/1 ($[V^{IV}O]^{2+}$ 1 mM), blue line; $[V^{IV}O]^{2+}$/ma/AT1 1/1/1 ($[V^{IV}O]^{2+}$ 200 μM), red line; $[V^{IV}O]^{2+}$/ma/MeIm 1/1/1 ($[V^{IV}O]^{2+}$ 1 mM), green line.

*Full-MS investigations on **1** – **6**.*

Table S2. Identified species in deconvoluted MS^1 spectrum in **1** ([AT2 + $VO(dhp)_2$]). Proton subtraction is performed in a formal manner to achieve charge neutralization.

	species	$m_{exp.}$	$m_{calc.}$	error [ppm]
**1**	[AT2]	1045.5351	1045.5340	1.05
	[AT2 + VO - 2H]	1110.4568	1110.4572	-0.36
	[AT2 + VO_2 - H]	1127.4613	1127.4605	0.71
	[AT2 + VO(dhp) - 2H]	1248.5141	1248.5127	1.12
	[AT2 + $VO(dhp)_2$]	1388.5854	1388.5830	1.73
	[AT2 + O]	1061.5294	1061.5289	0.47
	[AT2 + 2O]	1077.5245	1077.5238	0.65
	[AT2 + 2O + H_2O]	1095.5356	1095.5344	1.10
	[AT2 + O + VO_2 - H]	1143.4563	1143.4549	1.22
	[AT2 + VO_2(dhp)]	1266.5247	1266.5233	1.11
	[AT2 + VO(dhp) + VO_2 - 2H]	1331.4478	1331.4469	0.68
	[AT2 + 2VO(dhp) - 3H] (0.85)	1452.5007	1452.4993	0.96
	[AT2 + 2VO(dhp) - 2H] (0.15)	1453.5061	1453.5031	2.06
	[AT2 + O + 2VO(dhp) - 2H] (0.25)	1469.5033	1469.5019	0.95
	[AT2 + VO(dhp) + VO_2(dhp) - H] (0.75)	1470.5112	1470.5088	1.63
	[AT2 + $VO(dhp)_2$ + 2VO_2 - 2H]	1552.4377	1552.4358	1.22
	[2AT2 + VO(dhp) - H]	2295.0585	2295.0551	1.48
	[2AT2 + VO_2(dhp)]	2312.0610	2312.0578	1.38

Table S3. Identified species in deconvoluted MS[1] spectrum for **2** ([AT1 + $VO(dhp)_2$]). Proton subtraction is performed in a formal manner to achieve charge neutralization.

	species	$m_{exp.}$	$m_{calc.}$	error [ppm]
**2**	[AT1]	1295.6762	1295.6769	-0.54
	[AT1 + VO - 3H]	1359.5920	1359.5924	-0.29
	[AT1 + VO_2 - H]	1377.6020	1377.6028	-0.58
	[AT1 + VO(dhp) - 2H]	1498.6553	1498.6557	-0.27
	[AT1 + O]	1311.6707	1311.6719	-0.91
	[AT1 + 2O]	1327.6656	1327.6668	-0.90
	[AT1 + 2O + H_2O]	1345.6768	1345.6773	-0.37
	[AT1 + O + VO_2 - H]	1393.5974	1393.5978	-0.29
	[AT1 + 2O + VO_2 - H]	1409.5918	1409.5928	-0.70
	[AT1 + O + VO_2(dhp)]	1532.6606	1532.6603	0.20
	[AT1 + 2O + VO_2(dhp)]	1548.6546	1548.6559	-0.84
	[AT1 + VO_2 + VO(dhp) - 3H] (0.8)	1580.5808	1580.5823	-0.95
	[AT1 + VO_2 + VO(dhp) - 2H] (0.2)	1581.5860	1581.5863	-0.19
	[AT1 + 2O + VO + VO(dhp) - 3H]	1596.5761	1596.5774	-0.81
	[AT1 + 2VO(dhp) - 3H]	1702.6400	1702.6417	-1.00
	[AT1 + O + 2VO(dhp) - 2H] (0.9)	1719.6441	1719.6450	-0.52
	[AT1 + VO(dhp) + VO_2(dhp) - H] (0.1)	1720.6491	1720.6484	0.41
	[2AT1 + VO_2(dhp)]	2813.3415	2813.3451	-1.28

Table S4. Identified species in deconvoluted MS[1] spectrum for **3** ([AT2 + $VO(ma)_2$]). Proton subtraction is performed in a formal manner to achieve charge neutralization.

	species	$m_{exp.}$	$m_{calc.}$	error [ppm]
**3**	[AT2]	1045.5346	1045.5340	0.57
	[AT2 + VO - 2H]	1110.4577	1110.4566	0.99
	[AT2 + VO_2 - H]	1127.4604	1127.4605	-0.09
	[AT2 + VO(ma) - 2H]	1235.4823	1235.4811	0.97
	[AT2 + $VO(ma)_2$ - H]	1361.5143	1361.5129	1.03
	[AT2 + O]	1061.5295	1061.5289	0.57
	[AT2 + 2O]	1077.5247	1077.5238	0.84
	[AT2 + 2O + H_2O]	1095.5357	1095.5344	1.19
	[AT2 + O + VO_2 - H]	1143.4556	1143.4549	0.61
	[AT2 + 2VO - 5H]	1174.3735	1174.3726	0.77
	[AT2 + 2VO + H_2O - 5H]	1192.3844	1192.3836	0.67
	[AT2 + O + VO(ma) - 2H]	1251.4767	1251.4753	0.56
	[AT2 + O + VO_2(ma)]	1269.4879	1269.4859	1.58
	[AT2 + VO(ma) + VO_2 - 2H]	1318.4155	1318.4149	0.46
	[AT2 + 2VO(ma) - 3H] (0.55)	1426.4369	1426.4362	0.49
	[AT2 + 2VO(ma) - 2H] (0.45)	1427.4443	1427.4422	1.47
	[AT2 + 2VO(ma) + O - 2H] (0.85)	1443.4400	1443.4388	0.83
	[AT2 + VO(ma) + VO_2(ma) - H] (0.15)	1444.4453	1444.4427	1.80
	[AT2 + 2VO(ma) + VO_2 - 3H]	1509.3698	1509.3706	-0.53
	[AT2 + VO(ma) + VO_2(ma) + VO_2 - 2H] (0.9)	1526.3734	1526.3728	0.39
	[AT2 + 2VO(ma) + VO_2 + H_2O - 3H] (0.1)	1527.3765	1527.3767	-0.13
	[2AT2 + VO_2 - H]	2172.9943	2172.9945	-0.09

Table S5. Identified species in deconvoluted MS[1] spectrum for **4** ([AT1 + $VO(ma)_2$]). Proton subtraction is performed in a formal manner to achieve charge neutralization.

	species	$m_{exp.}$	$m_{calc.}$	error [ppm]
**4**	[AT1]	1295.6772	1295.6769	0.23
	[AT1 + VO - 2H]	1360.5991	1360.6002	-0.81
	[AT1 + VO_2 - H]	1377.6032	1377.6029	0.22
	[AT1 + VO(ma) - 2H]	1485.6245	1485.6241	0.27
	[AT1 + O]	1311.6714	1311.6719	-0.38
	[AT1 + 2O]	1327.6666	1327.6668	-0.15
	[AT1 + 2O + H_2O]	1345.6778	1345.6773	0.37
	[AT1 + VO_2 + O - H]	1393.5984	1393.5971	0.93
	[AT1 + O + VO_2(ma)]	1519.6299	1519.6295	0.26
	[AT1 + VO(ma) + VO - 4H] (0.1)	1550.5481	1550.5478	0.19
	[AT1 + VO(ma) + VO - 3H] (0.9)	1551.5541	1551.5547	-0.39
	[AT1 + VO_2 + VO(ma) - 2H]	1568.5568	1568.5570	-0.13
	[AT1 + VO + VO(ma) + 2O - 3H]	1583.5454	1583.5453	0.06
	[AT1 + O + VO_2 + VO_2(ma) - H]	1601.5554	1601.5546	0.50
	[AT1 + 2VO(ma) - 3H] (0.9)	1676.5792	1676.5790	0.12
	[AT1 + 2VO(ma) - 2H] (0.1)	1677.5841	1677.5825	0.95
	[AT1 + VO(ma) + VO_2(ma) - 2H] (0.9)	1693.5820	1693.5811	0.53
	[AT1 + VO(ma) + VO_2(ma) - H] (0.1)	1694.5875	1694.5849	1.53

Table S6. Identified species in deconvoluted MS[1] spectrum for **5** ([AT2 + $VO(pic)_2$]). Proton subtraction is performed in a formal manner to achieve charge neutralization.

	species	$m_{exp.}$	$m_{calc.}$	error [ppm]
**5**	[AT2]	1045.5332	1045.5340	-0.77
	[AT2 + VO - 2H]	1110.4565	1110.4572	-0.63
	[AT2 + VO_2 - H]	1127.4597	1127.4599	-0.18
	[AT2 + VO(pic) - H]	1233.4889	1233.4896	-0.57
	[AT2 + $VO(pic)_2$]	1356.5212	1356.5213	-0.07
	[AT2 + O]	1061.5284	1061.5289	-0.47
	[AT2 + 2O]	1077.5233	1077.5283	-0.46
	[AT2 + 2O + H_2O]	1095.5342	1095.5344	-0.18
	[AT2 + O + VO_2 - H]	1143.4545	1143.4549	-0.35
	[AT2 + VO + VO_2 - 3H]	1192.3832	1192.3836	-0.34
	[AT2 + VO_2(pic) + H]	1250.4916	1250.4920	-0.32
	[AT2 + O + VO_2(pic)]	1266.4866	1266.4870	-0.32
	[AT2 + VO(pic) + VO - 3H]	1298.4121	1298.4125	-0.31
	[AT2 + 2VO(pic) - 2H]	1421.4438	1421.4448	-0.70
	[AT2 + VO(pic) + VO_2(pic) - H]	1438.4468	1438.4481	-0.90
	[AT2 + 2VO_2(pic) - H]	1454.4416	1454.4422	-0.41
	[AT2 + VO(pic) + $VO(pic)_2$ - H]	1544.4764	1544.4759	0.32
	[AT2 + VO_2(pic) + $VO(pic)_2$]	1561.4784	1561.4792	-0.51
	[AT2 + VO_2(pic) + $VO_2(pic)_2$]	1577.4735	1577.4748	-0.82
	[AT2 + 2$VO(pic)_2$]	1667.5079	1667.5085	-0.36
	[AT2 + 2$VO(pic)_2$ + VO - 2H]	1732.4309	1732.4316	-0.40
	[AT2 + 2$VO(pic)_2$ + VO(pic) - H]	1855.4632	1855.4629	0.16
	[2AT2 + VO_2 - H]	2172.9943	2172.9945	-0.09

Table S7. Identified species in deconvoluted MS[1] spectrum for **6** ([AT1 + $VO(pic)_2$]). Proton subtraction is performed in a formal manner to achieve charge neutralization.

	species	$m_{exp.}$	$m_{calc.}$	error [ppm]
**6**	[AT1]	1295.6773	1295.6769	0.31
	[AT1 + VO - 2H]	1360.6003	1360.6002	0.07
	[AT1 + VO_2 - H]	1377.6018	1377.6029	-0.80
	[AT1 + VO(pic) - H]	1483.6324	1483.6322	0.13
	[AT1 + $VO(pic)_2$]	1606.6647	1606.6642	0.31
	[AT1 + O]	1311.6714	1311.6719	-0.38
	[AT1 + 2O]	1327.6666	1327.6668	-0.15
	[AT1 + 2O + H_2O]	1345.6778	1345.6773	0.37
	[AT1 + O + VO_2 - H]	1393.5981	1393.5971	0.72
	[AT1 + VO_2(pic)]	1500.6347	1500.6350	-0.20
	[AT1 + O + VO_2(pic)]	1516.6300	1516.6299	0.07
	[AT1 + VO(pic) + VO - 3H]	1548.5556	1548.5562	-0.39
	[AT1 + VO_2 + VO(pic) - 2H]	1565.5567	1565.5582	-0.96
	[AT1 + O + VO_2 + VO(pic) - 2H]	1581.5535	1581.5531	0.25
	[AT1 + $VO(pic)_2$ - H_2O]	1589.6620	1589.6615	0.31
	[AT1 + $VO(pic)_2$]	1606.6647	1606.6642	0.31
	[AT1 + O + $VO_2(pic)_2$ - H]	1639.6624	1639.6609	0.91
	[AT1 + 2VO(pic) - 2H]	1671.5878	1671.5881	-0.18
	[AT1 + VO(pic) + VO_2(pic) - H]	1688.5898	1688.5901	-0.18
	[AT1 + O + VO(pic) + VO_2(pic) - H]	1704.5852	1704.5851	0.06
	[AT1 + 2O + 2VO_2(pic)]	1737.5832	1737.5819	0.75
	[AT1 + $VO(pic)_2$ + VO(pic) - H]	1794.6202	1794.6190	0.67
	[AT1 + 2$VO(pic)_2$]	1918.6554	1918.6544	0.52

MS^2 HCD-fragmentation

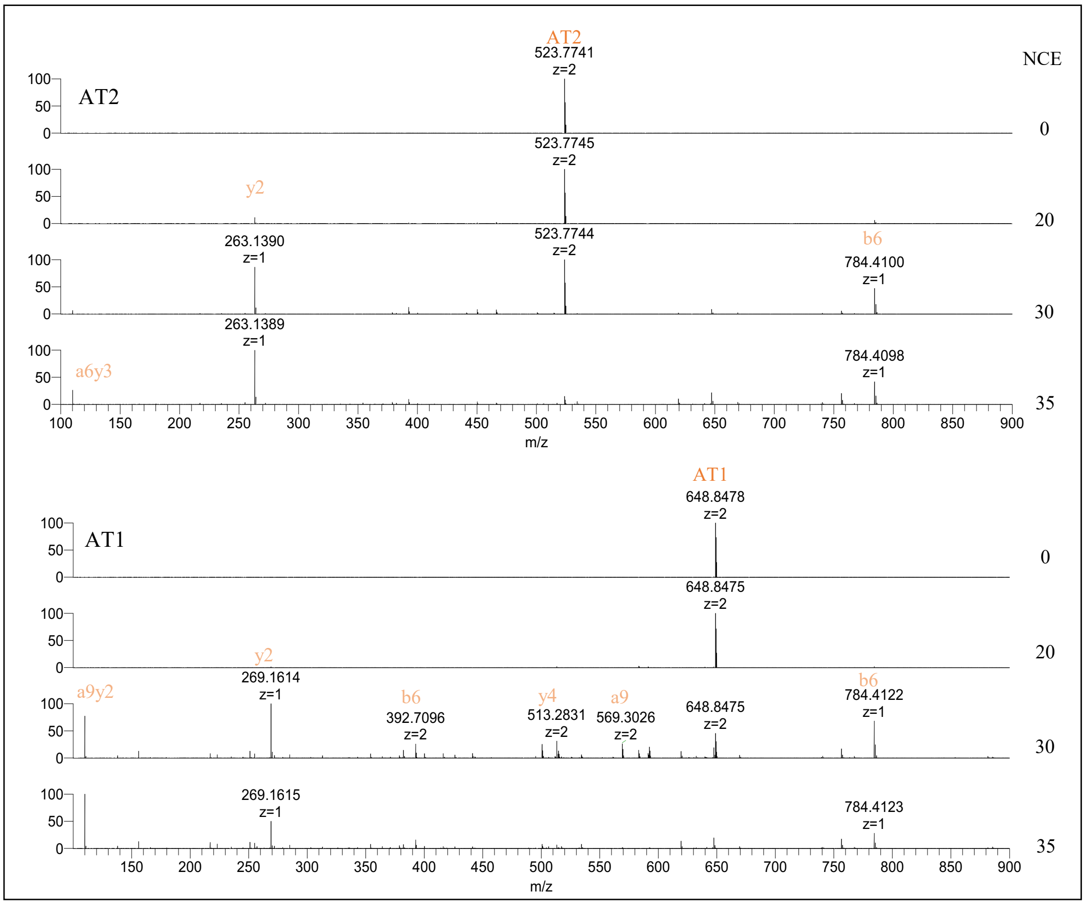

Figure S5. Fragmentation behavior of AT2 (top) and AT1 (bottom) precursor ions at NCE 0-35

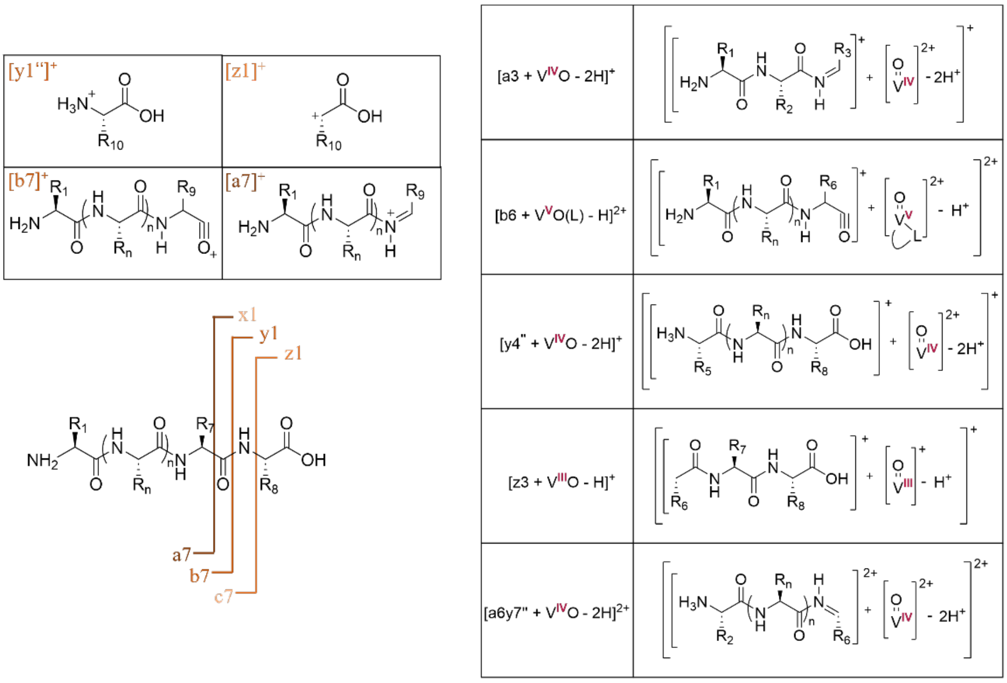

Figure S6. Left: Main backbone HCD-fragmentation patterns of peptides; Right: schematic examples of fragments and the proposed procedure to monitor the formal oxidation state of vanadium.

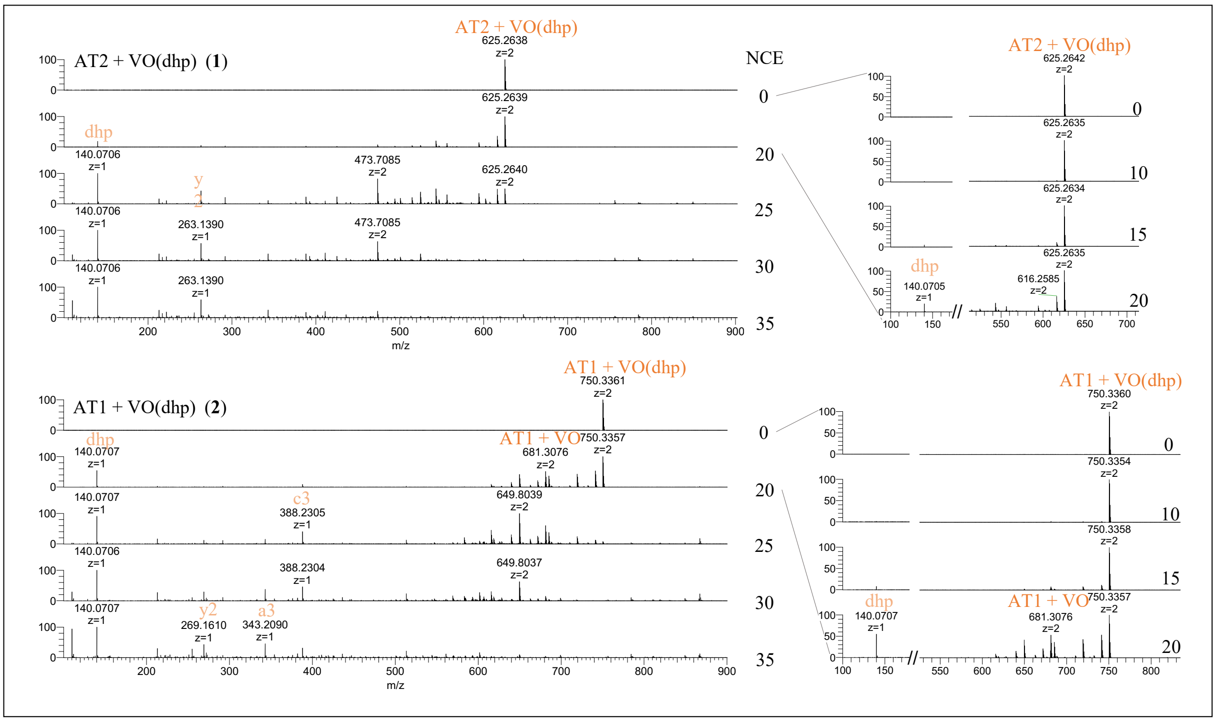

Figure S7. Fragmentation behavior of [AT2 + VO(dhp)] (top) and [AT1 + VO(dhp)] (bottom) precursor ions at NCE 0-35.

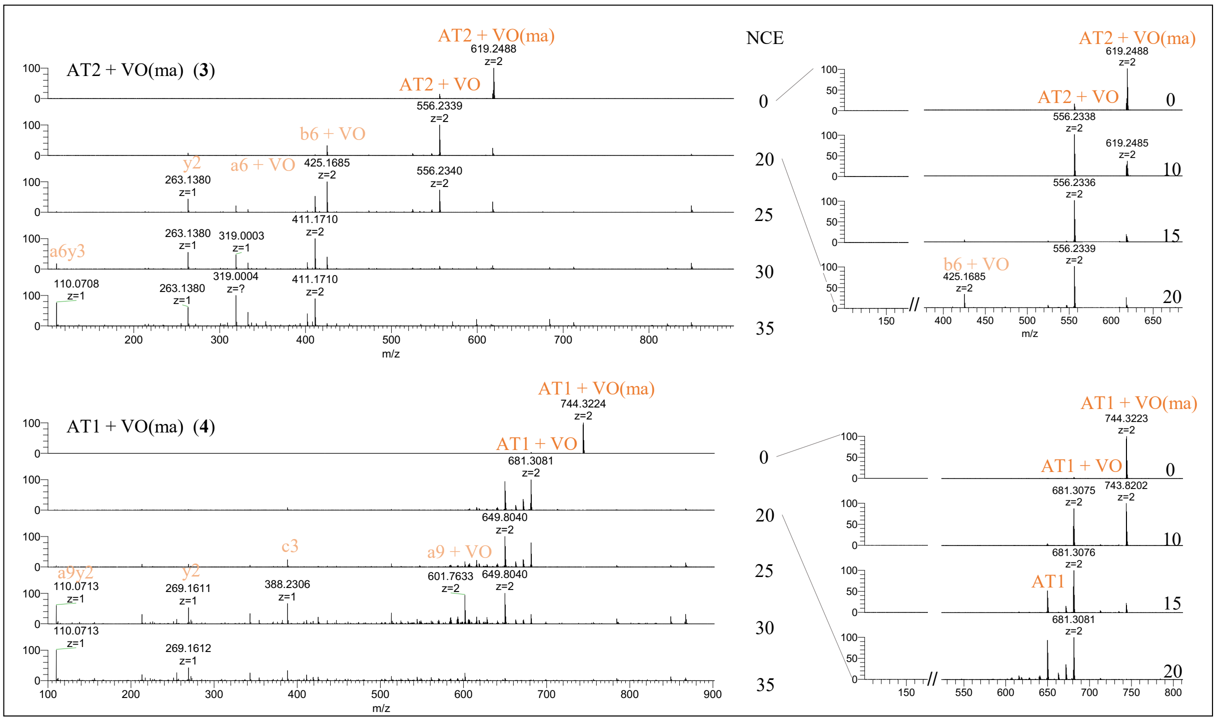

Figure S8. Fragmentation behavior of [AT2 + VO(ma)] (top) and [AT1 + VO(ma)] (bottom) precursor ions at NCE 0-35.

1. Fragmentation of [AT2 + V^{IV}O(dhp) + H]$^{2+}$ / [AT2 + V^{V}O(dhp)]$^{2+}$ (ratio: 0.1 / 0.9)

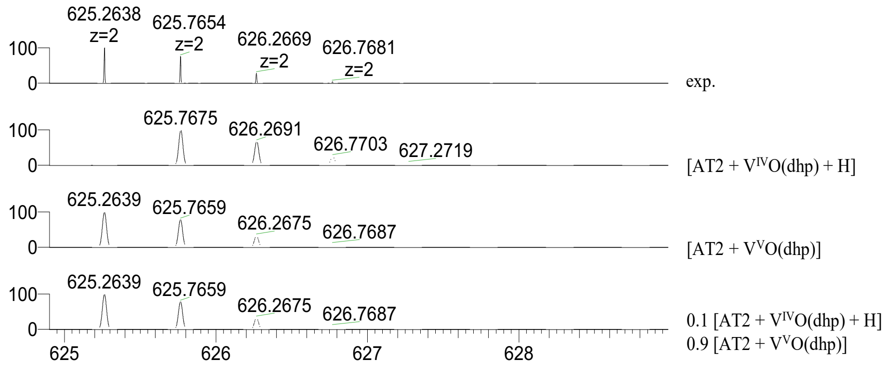

Figure S9. Isotopic distribution of the precursor ion of **1** ([AT2 + V^{IV}O(dhp)]) at NCE 0 in the experimental spectrum (Top spectrum); calculated pattern for [AT2 + V^{IV}O(dhp) + H]$^{2+}$ (Second spectrum); calculated pattern for [AT2 + V^{V}O(dhp)]$^{2+}$ (Third spectrum) and calculated spectrum for a 1/9 ratio of V^{IV} and V^{V} (Bottom).

Table S8: Identified metalated fragments after fragmentation of [AT2 + VO(dhp)]$^{2+}$ precursor ion in **1** (NCE 20-35 average).

species	$m_{exp.}$	$m_{calc.}$	error [ppm]	Ox. state	Rel. Ab. /%
[AT2 + VO(dhp)]$^{2+}$	625.2638	625.2639	-0.16	5	37.18
[AT2 + VO(dhp) + H]$^{2+}$	625.7654	625.7657	-0.48	4	26.13
N-terminal fragments					
[a1 + VO(dhp) - H]$^{+}$	292.0257	292.0259	-0.68	4	12.78
[a2 + VO - 2H]$^{+}$	309.0637	309.0635	0.65	4	1.48
[b2 + VO - 2H]$^{+}$	337.0586	337.0584	0.59	4	1.59
[a3 + VO - 2H]$^{+}$	408.1321	408.1319	0.49	4	4.08
[b3 + VO - 2H]$^{+}$	436.1269	436.1272	-0.69	4	1.20
[a4 + VO - 2H]$^{+}$	571.1954	571.1954	0.00	4	1.63
[b4 + VO - 2H]$^{+}$	599.1905	599.1903	0.33	4	3.02
[a5 + VO - 2H]$^{+}$	684.2796	684.2793	0.44	4	2.62
[b5 + VO - 2H]$^{+}$	712.2751	712.2744	0.98	4	1.58
[a6 + VO - 2H]$^{+}$	821.3383	821.3384	-0.12	4	1.58
[a6 + VO - H]$^{2+}$	411.1729	411.1728	0.24	4	**18.56**
[a6 + VO(dhp) - H]$^{2+}$	480.2006	480.2006	0.00	5	0.75
[b6 + VO - 3H]$^{+}$ (0.15)	848.3251	848.3255	-0.47	5	0.57
[b6 + VO - 2H]$^{+}$ (0.85)	849.3329	849.3328	0.12	4	5.04
[b6 + VO - H]$^{2+}$	425.1704	425.1701	0.71	4	**10.51**
[b6 + VO(dhp)]$^{2+}$	494.1982	494.198	0.40	4	6.96
[a7 + VO(dhp)]$^{2+}$	529.2307	529.2309	-0.38	4	0.48
[a7 + VO(dhp) + H]$^{2+}$	529.7345	529.7348	-0.57	3	0.89
[a8 + VO - 2H]$^{2+}$	532.7295	532.7296	-0.18	5	1.27
[a8 + VO(dhp) - H]$^{2+}$	602.2614	602.2609	0.83	5	5.34
[b8 + VO - 3H]$^{+}$ (0.8)	1092.4462	1092.4462	0.00	5	1.27
[b8 + VO - 2H]$^{+}$ (0.2)	1093.4514	1093.4504	0.91	4	1.03
[b8 + VO - 2H]$^{2+}$	546.7270	546.7272	-0.37	5	5.39
[b8 + VO(dhp) - H]$^{2+}$	616.2587	616.2586	0.16	5	18.99
[AT2 + VO - H]$^{2+}$ (0.3)	555.7322	555.7324	-0.36	5	2.53
[AT2 + VO]$^{2+}$ (0.7)	556.2361	556.2362	-0.18	4	13.11
C-terminal fragments					
[y3'' + VO - 2H]$^{+}$	465.1212	465.1210	0.43	4	0.49
[y4'' + VO - 2H]$^{+}$ (0.6)	578.2053	578.2052	0.17	4	0.90
[y4'' + VO - H]$^{+}$ (0.4)	579.2132	579.2131	0.17	3	0.86

[y6'' + VO - 2H]$^{+}$	840.3366	840.3370	-0.48	4	0.24
[y7'' + VO - H]$^{2+}$	498.7227	498.7227	0.00	4	0.67
[y7'' + VO(dhp) - H]$^{2+}$	567.7504	567.7504	0.00	5	1.47
internal fragments					
[a6y7'' + VO - 2H]$^{2+}$	353.6593	353.6593	0.00	4	1.91
[b6y7'' + VO - 2H]$^{2+}$	367.6568	367.6567	0.27	4	0.45
[b6y5'' + VO - 3H]$^{+}$	479.1369	479.1368	0.21	4	0.58
[a7y6'' + VO(dhp) - 2H]$^{+}$	786.3251	786.3266	-1.91	4	3.29

2. Fragmentation of [AT1 + V^{IV}O(dhp) + H]$^{2+}$ / [AT1 + V^{V}O(dhp)]$^{2+}$ (ratio: 0.05 / 0.95)

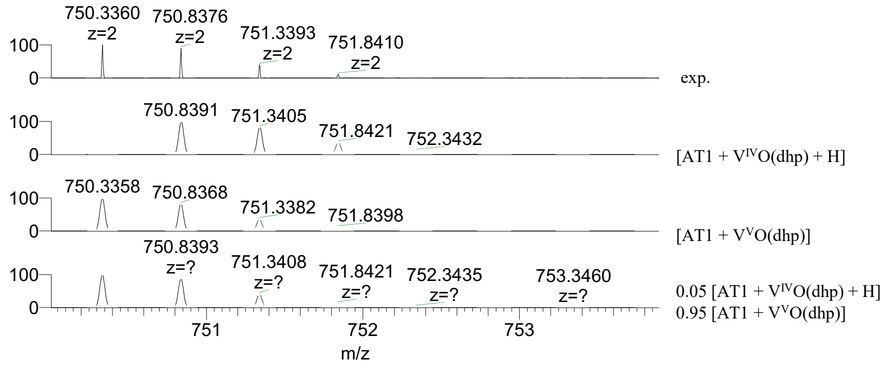

Figure S10. Isotopic distribution of the precursor ion of **2** ([AT1 + V^{IV}O(dhp)]) at NCE 0 in the experimental spectrum (Top spectrum); calculated pattern for [AT1 + V^{IV}O(dhp) + H]$^{2+}$ (Second spectrum); calculated pattern for [AT1 + V^{V}O(dhp)]$^{2+}$ (Third spectrum) and calculated spectrum for a 0.5/9.5 ratio of V^{IV} and V^{V} (Bottom).

Table S9: Identified metalated fragments after fragmentation of [AT1 + VO(dhp)] precursor in **2** (NCE 20-35 average).

species	$m_{exp.}$	$m_{calc.}$	error [ppm]	Ox. state	Rel. Ab. /%
[AT1 + VO(dhp)]$^{2+}$	750.3360	750.3358	0.27	5	66.26
[AT1 + VO(dhp) + H]$^{2+}$	750.8376	750.8391	-2.00	4	54.60
N-terminal fragments					
[a1 + VO(dhp) - H]$^{+}$	292.0260	292.0257	1.03	4	9.23
[a2 + VO - 2H]$^{+}$	309.0638	309.0635	0.97	4	0.57
[b2 + VO - 2H]$^{+}$	337.0587	337.0586	0.30	4	0.74
[a3 + VO - 2H]$^{+}$	408.1321	408.1323	0.49	4	2.31
[b3 + VO - 2H]$^{+}$	436.1273	436.1272	0.23	4	0.95
[a4 + VO - 2H]$^{+}$	571.1958	571.1952	1.05	4	0.59
[b4 + VO - 2H]$^{+}$	599.1906	599.1903	0.50	4	1.18
[a5 + VO - 2H]$^{+}$	684.2801	684.2793	1.17	4	0.23
[b5 + VO - 2H]$^{+}$	712.2746	712.2744	0.28	4	0.23
[a6 + VO - 2H]$^{+}$	821.3387	821.3389	-0.24	4	0.58
[a6 + VO - H]$^{2+}$	411.1730	411.1728	0.49	4	1.87
[b6 + VO - 3H]$^{+}$ (0.05)	848.3260	848.3260	0.00	5	0.34
[b6 + VO - 2H]$^{+}$ (0.95)	849.3345	849.3333	1.41	4	4.65
[b6 + VO - H]$^{2+}$	425.1705	425.1701	0.94	4	3.13
[b6 + VO(dhp) - H]$^{2+}$	494.1983	494.1979	0.81	5	0.95
[b7 + VO - H]$^{2+}$	473.6968	473.6966	0.42	4	0.39
[a8 + VO - H]$^{2+}$	533.2335	533.2334	0.19	4	1.31
[b8 + VO - 2H]$^{+}$	1093.4545	1093.4545	0.00	4	1.31

[a9 + VO - H]$^{2+}$	601.7630	601.7630	0.00	4	**14.67**
[a9 + VO(dhp) + H]$^{2+}$	671.7989	671.7987	0.30	3	21.85
[b9 + VO - H]$^{2+}$	615.7605	615.7605	0.00	4	**7.62**
[AT1 + VO - H]$^{2+}$ (0.3)	680.8042	680.8038	0.59	5	12.37
[AT1 + VO]$^{2+}$ (0.7)	681.3079	681.3076	1.32	4	51.54
C-terminal fragments					
[y4'' + VO - 2H]$^{+}$	578.2055	578.2050	0.86	4	1.83
[y5'' + VO - 2H]$^{+}$	715.2647	715.2644	0.42	4	0.76
[z5 + VO - H]$^{+}$	699.2456	699.2452	0.57	3	4.76
[y9'' + VO - H]$^{2+}$	623.7942	623.7942	0.00	4	1.29
[y9'' + VO(dhp) - H]$^{2+}$	692.8223	692.8219	0.58	5	0.53
internal fragments					
[a8y6'' + VO - 2H]$^{+}$	533.2078	533.2076	0.38	3	1.40
[b8y6'' + VO - 2H]$^{+}$	561.2027	561.2025	0.36	3	3.35
[a9y4'' + VO - 3H]$^{+}$	419.1159	419.1157	0.48	4	1.49
[b9y4'' + VO - 3H]$^{+}$	447.1109	447.1106	0.67	4	2.61
[a9y9'' + VO - 2H]$^{2+}$	544.2496	544.2494	0.37	4	2.28
[a9z9 + VO - 2H]$^{2+}$	535.7360	535.7361	-0.19	4	0.13
[b9y9'' + VO - 2H]$^{2+}$	558.2471	558.2472	-0.18	4	0.42
[b9y9'' + VO(dhp)]$^{2+}$	628.2828	628.2825	0.48	3	6.99
[a6y7'' + VO - 3H]$^{+}$	451.1423	451.1421	0.44	4	0.21
[b6y7'' + VO - 3H]$^{+}$	479.1371	479.1368	0.63	4	0.44
[a9y8'' + VO - 3H]$^{+}$	931.3911	931.3904	0.75	4	0.20
[a9y5'' + VO - 3H]$^{+}$	556.1748	556.1745	0.54	4	0.31
[a6y9'' + VO - 2H]$^{2+}$	353.6594	353.6593	0.28	4	0.11

3. Fragmentation of [AT2 + V^{IV}O(ma) + H]$^{2+}$ / [AT2 + V^{V}O(ma)]$^{2+}$ (ratio: 0.15 / 0.85)

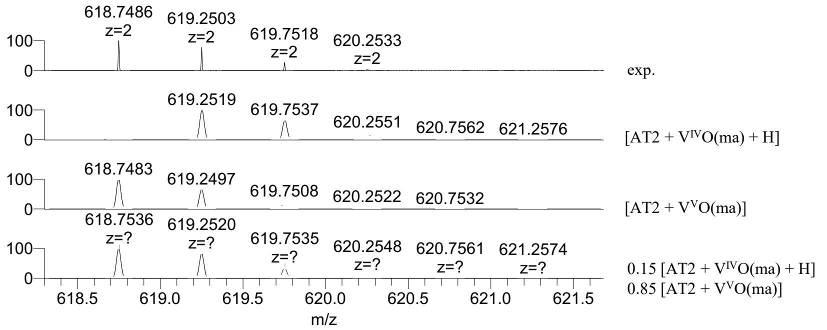

Figure S11. Isotopic distribution of the precursor ion of **3** ([AT2 + V^{IV}O(ma)]) in MS^1 spectrum in the experimental spectrum (Top spectrum); calculated pattern for [AT2 + V^{IV}O(ma) + H]$^{2+}$ (Second spectrum); calculated pattern for [AT2 + V^{V}O(ma)]$^{2+}$ (Third spectrum) and calculated spectrum for a 1.5/8.5 ratio of V^{IV} and V^{V} (Bottom).

Table S10. Identified metalated fragments after fragmentation of [AT2 + VO(ma)]$^{2+}$ precursor ion in **3** (NCE 20-35 average).

species	$m_{exp.}$	$m_{calc.}$	error [ppm]	Ox. state	Rel. Ab. /%
[AT2 + VO(ma)]$^{2+}$	618.7490	618.7481	1.45	5	20.84
[AT2 + VO(ma) + H]$^{2+}$	619.2506	619.2496	1.61	4	13.45
N-terminal fragments					
[a1 + VO(ma) - H]$^{+}$	278.9945	278.9942	1.07	4	5.19
[a2 + VO - 2H]$^{+}$	309.0638	309.0635	0.97	4	0.87

[b2 + VO - 2H]$^{+}$	337.0588	337.0586	0.59	4	1.05
[a3 + VO - 2H]$^{+}$	408.1325	408.1319	1.47	4	2.66
[b3 + VO - 2H]$^{+}$	436.1275	436.1270	1.15	4	1.23
[a4 + VO - 2H]$^{+}$	571.1965	571.1952	2.28	4	0.61
[b4 + VO - 2H]$^{+}$	599.1914	599.1903	1.84	4	1.76
[a5 + VO - 2H]$^{+}$	684.2804	684.2793	1.61	4	1.21
[b5 + VO - 3H]$^{+}$ (0.4)	711.2672	711.2666	0.84	5	0.88
[b5 + VO - 2H]$^{+}$ (0.6)	712.2748	712.2744	0.56	4	1.60
[a6 + VO - 2H]$^{+}$	821.3388	821.3384	0.49	4	0.89
[a6 + VO - H]$^{2+}$	411.1732	411.1727	1.22	4	**27.84**
[b6 + VO - 3H]$^{+}$ (0.3)	848.3260	848.3255	0.59	5	2.38
[b6 + VO - 2H]$^{+}$ (0.7)	849.3337	849.3333	0.47	4	7.22
[b6 + VO - 2H]$^{2+}$ (0.2)	424.6667	424.6664	0.71	5	5.79
[b6 + VO - H]$^{2+}$ (0.8)	425.1707	425.1703	0.94	4	**29.12**
[b7 + VO - H]$^{2+}$	473.6973	473.6966	1.48	4	1.13
[b7 + VO(ma) - H]$^{2+}$	536.2092	536.2086	1.12	5	0.34
[a8 + VO(ma) - H]$^{2+}$	595.7462	595.7457	0.84	5	1.72
[b8 + VO - 2H]$^{2+}$	546.7277	546.7272	0.91	5	58.71
[b8 + VO(ma) - H]$^{2+}$	609.7438	609.7428	1.64	5	9.73
[AT2 + VO - H]$^{2+}$ (0.5)	555.7329	555.7323	1.08	5	20.50
[AT2 + VO]$^{2+}$ (0.5)	556.2364	556.2362	0.34	4	40.60
C-terminal fragments					
[y3'' + VO - 2H]$^{+}$	465.1216	465.1210	1.29	4	0.46
[z3 + VO - H]$^{+}$	449.1029	449.1027	0.45	3	0.72
[y4'' + VO - 2H]$^{+}$ (0.3)	578.2060	578.2052	1.38	4	0.40
[y4'' + VO - H]$^{+}$ (0.7)	579.2139	579.2131	1.38	3	1.33
[y6'' + VO - 2H]$^{+}$	840.3372	840.3370	0.24	4	0.13
[y7'' + VO - H]$^{2+}$	498.7225	498.7227	-0.4	4	0.73
[y7'' + VO(ma) - H]$^{2+}$	561.2352	561.2345	1.25	5	0.53
internal fragments					
[a6y7'' + VO - 2H]$^{2+}$	353.6595	353.6593	0.57	4	1.00
[b6y7'' + VO - 2H]$^{2+}$	367.6570	367.6568	0.54	4	0.36
[a6y5'' + VO - 3H]$^{+}$	451.1426	451.1421	1.11	4	0.27
[b6y5'' + VO - 3H]$^{+}$	479.1372	479.1368	0.83	4	0.30

4. Fragmentation of [AT1 + V^{IV}O(ma) + H]$^{2+}$ / [AT1 + V^{V}O(ma)]$^{2+}$ (ratio: 0.2 / 0.8)

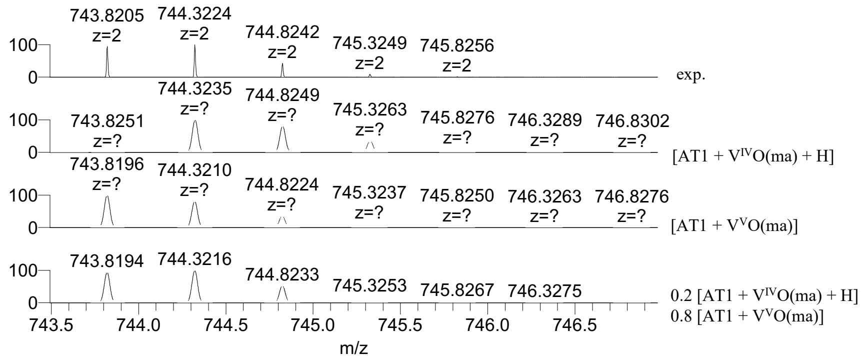

Figure S12. Isotopic distribution of the precursor ion of **4** ([AT1 + V^{IV}O(ma)]) at NCE 0 in the experimental spectrum (Top spectrum); calculated pattern for [AT1 + V^{IV}O(ma) + H]$^{2+}$ (Second spectrum); calculated pattern for [AT1 + V^{V}O(ma)]$^{2+}$ (Third spectrum) and calculated spectrum for a 2/8 ratio of V^{IV} and V^{V} (Bottom).

Table S11. Identified metalated fragments after fragmentation of [AT1 + VO(ma)]$^{2+}$ precursor ion in **4** (NCE 20-35 average).

species	$m_{exp.}$	$m_{calc.}$	error [ppm]	Ox. state	Rel. Ab. /%
[AT1 + VO(ma)]$^{2+}$	743.8200	743.8196	0.54	5	0.59
[AT1 + VO(ma) + H]$^{2+}$	744.3215	744.3216	-0.13	4	0.47
N-terminal fragments					
[a1 + VO(ma) - H]$^{+}$	278.9945	278.9941	1.43	4	0.49
[a2 + VO - 2H]$^{+}$	309.0640	309.0636	1.29	4	0.12
[b2 + VO - 2H]$^{+}$	337.0589	337.0584	1.48	4	1.02
[a3 + VO - 2H]$^{+}$	408.1325	408.1321	0.98	4	2.28
[b3 + VO - 2H]$^{+}$	436.1274	436.1272	0.46	4	1.86
[a4 + VO - 2H]$^{+}$	571.1960	571.1954	1.05	4	0.80
[b4 + VO - 2H]$^{+}$	599.1910	599.1903	1.17	4	1.78
[a5 + VO - 2H]$^{+}$	684.2803	684.2795	1.17	4	0.34
[b5 + VO - 2H]$^{+}$	712.2751	712.2740	1.54	4	0.41
[a6 + VO - 2H]$^{+}$	821.3396	821.3384	1.46	4	0.76
[a6 + VO - H]$^{2+}$	411.1732	411.1728	0.97	4	3.71
[b6 + VO - 3H]$^{+}$ (0.05)	848.3261	848.3260	0.12	5	0.33
[b6 + VO - 2H]$^{+}$ (0.95)	849.3344	849.3333	1.30	4	7.39
[b6 + VO - H]$^{2+}$	425.1707	425.1703	0.94	4	5.19
[b7 + VO - H]$^{2+}$	473.6971	473.6967	0.84	4	0.53
[a8 + VO - H]$^{2+}$	533.2337	533.2334	0.56	4	1.92
[b8 + VO - H]$^{2+}$	547.2310	547.2309	0.18	4	1.53
[b8 + VO - 2H]$^{+}$	1093.4550	1093.4550	0.00	4	2.32
[a9 + VO - H]$^{2+}$	601.7634	601.7630	0.66	4	**25.01**
[b9 + VO - H]$^{2+}$	615.7607	615.7603	0.65	4	**14.98**
[AT1 + VO - H]$^{2+}$ (0.2)	680.8045	680.8038	1.03	5	14.50
[AT1 + VO]$^{2+}$ (0.8)	681.3081	681.3073	1.17	4	85.96
C-terminal fragments					
[y4“ + VO - 2H]$^{+}$	578.2059	578.2052	1.21	4	2.42
[y5“ + VO - 2H]$^{+}$	715.2648	715.2644	0.56	4	0.76
[z5 + VO - H]$^{+}$	699.2461	699.2452	1.29	3	2.06
[y8“ + VO - 2H]$^{+}$	1073.4777	1073.4772	0.47	4	1.74
[y8“ + VO - H]$^{2+}$	537.2426	537.2422	0.74	4	0.78
[y9“ + VO - H]$^{2+}$	623.7946	623.7942	0.64	4	1.97
internal fragments					
[a8y6“ + VO - 2H]$^{+}$	533.2080	533.2076	0.75	3	1.33
[b8y6“ + VO - 2H]$^{+}$	561.2030	561.2025	0.89	3	2.70
[a9y4“ + VO - 3H]$^{+}$	419.1161	419.1157	0.95	4	2.38
[b9y4“ + VO - 3H]$^{+}$	447.1110	447.1106	0.89	4	3.55
[a9y9“ + VO - 2H]$^{2+}$	544.2498	544.2494	0.73	4	3.45
[b9y9“ + VO - 2H]$^{2+}$	558.2474	558.2469	0.90	4	0.63
[a6y7“ + VO - 3H]$^{+}$	451.1425	451.1421	0.89	4	0.19
[b6y7“ + VO - 3H]$^{+}$	479.1373	479.1368	1.04	4	0.38
[a9y8“ + VO - 3H]$^{+}$	931.3916	931.3904	1.29	4	0.25
[a9y5“ + VO - 3H]$^{+}$	556.1751	556.1746	0.90	4	0.36
[a6y9“ + VO - 3H]$^{+}$	353.6598	353.6593	1.41	4	0.17

5. Fragmentation of [AT2 + V^{IV}O(pic) + H]$^{2+}$ / [AT2 + V^{V}O(pic)]$^{2+}$ (ratio: 0.5 / 0.5)

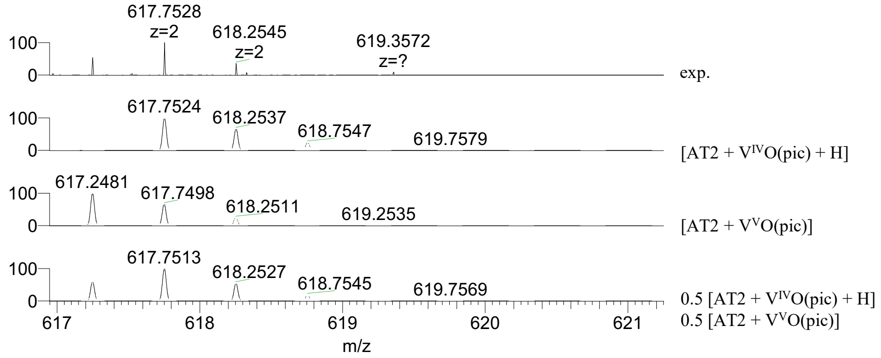

Figure S13. Isotopic distribution of the precursor ion of **5** ([AT2 + V^{IV}O(pic)]) at NCE 0 in the experimental spectrum (Top spectrum); calculated pattern for [AT2 + V^{IV}O(pic) + H]$^{2+}$ (Second spectrum); calculated pattern for [AT2 + V^{V}O(pic)]$^{2+}$ (Third spectrum) and calculated spectrum for a 1/1 ratio of V^{IV} and V^{V} (Bottom).

Table S12. Identified metalated fragments after fragmentation of [AT2 + VO(pic)] precursor in **5** (NCE 20-35 average).

species	$m_{exp.}$	$m_{calc.}$	error [ppm]	Ox. state	Rel. Ab. /%
[AT2 + VO(pic)]$^{2+}$	617.2493	617.2483	1.62	5	0.13
[AT2 + VO(pic) + H]$^{2+}$	617.7527	617.7522	0.81	4	0.18
N-terminal fragments					
[a2 + VO - 2H]$^{+}$	309.0642	309.0635	2.26	4	7.03
[b2 + VO - 2H]$^{+}$	337.0592	337.0586	1.78	4	6.87
[a3 + VO - 2H]$^{+}$	408.1328	408.1319	2.21	4	10.86
[b3 + VO - 2H]$^{+}$	436.1277	436.1270	1.61	4	4.87
[a4 + VO - 2H]$^{+}$	571.1965	571.1952	2.28	4	8.61
[b4 + VO - 2H]$^{+}$	599.1914	599.1903	1.84	4	13.71
[a5 + VO - 2H]$^{+}$	684.2806	684.2793	1.90	4	13.96
[b5 + VO - 2H]$^{+}$	712.2757	712.2744	1.83	4	8.29
[a6 + VO - 2H]$^{+}$	821.3398	821.3389	1.10	4	5.66
[a6 + VO - H]$^{2+}$	411.1735	411.1728	1.70	4	**93.57**
[b6 + VO - 2H]$^{+}$	849.3344	849.3333	1.30	4	21.41
[b6 + VO - H]$^{2+}$	425.1710	425.1701	2.12	4	**57.21**
[a7 + VO - H]$^{2+}$	459.7003	459.6993	2.18	4	0.56
[b7 + VO - H]$^{2+}$	473.6973	473.6967	1.27	4	1.12
[a8 + VO - 2H]$^{2+}$ (0.6)	532.7305	532.7296	1.69	5	1.95
[a8 + VO - H]$^{2+}$ (0.4)	533.2350	533.2334	3.00	4	2.86
[b8 + VO - 2H]$^{2+}$	546.7278	546.7270	1.46	5	12.98
[AT2 + VO - H]$^{2+}$ (0.3)	555.7330	555.7324	1.08	5	7.36
[AT2 + VO]$^{2+}$ (0.7)	556.2372	556.2362	1.80	4	74.75
C-terminal fragments					
[y3“ + VO - 2H]$^{+}$	465.1221	465.1210	2.36	4	0.25
[y4“ + VO - 2H]$^{+}$	578.2063	578.2052	1.90	4	0.91
[y7“ + VO - H]$^{2+}$	498.7238	498.7227	2.21	4	0.48
[z7 + VO - H]$^{+}$	490.7142	490.7133	1.83	3	0.26
internal fragments					
[a6y7“ + VO - 2H]$^{2+}$	353.6600	353.6594	1.70	4	6.37
[b6y7“ + VO - 2H]$^{2+}$	367.6574	367.6568	1.63	4	0.65
[a6y5“ + VO - 3H]$^{+}$	451.1429	451.1419	2.22	4	3.02
[b6y5“ + VO - 3H]$^{+}$	479.1379	479.1368	2.30	4	0.25

6. Fragmentation of [AT1 + V^{IV}O(pic) + H]$^{2+}$ / [AT1 + V^{V}O(pic)]$^{2+}$ (ratio: 0.95 / 0.05)

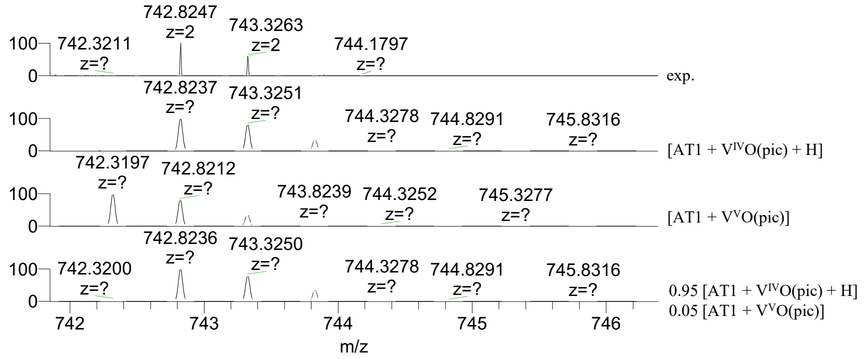

Figure S14. Isotopic distribution of the precursor ion of **6** ([AT1 + V^{IV}O(pic)]) at NCE 0 in the experimental spectrum (Top spectrum); calculated pattern for [AT1 + V^{IV}O(pic) + H]$^{2+}$ (Second spectrum); calculated pattern for [AT1 + V^{V}O(pic)]$^{2+}$ (Third spectrum) and calculated spectrum for a 1/1 mixture of V^{IV} and V^{V} (Bottom).

Table S13. Identified metalated fragments after fragmentation of [AT1 + VO(pic)]$^{2+}$ precursor ion in **6** (NCE 20-35 average).

species	$m_{exp.}$	$m_{calc.}$	error [ppm]	Ox. state	Rel. Ab. /%
[AT1 + VO(pic)]$^{2+}$	742.3209	742.3197	1.62	5	0.00
[AT1 + VO(pic) + H]$^{2+}$	742.8246	742.8237	1.21	4	0.00
N-terminal fragments					
[a2 + VO - 2H]$^{+}$	309.0641	309.0635	1.94	4	1.37
[b2 + VO - 2H]$^{+}$	337.0591	337.0586	1.48	4	1.43
[a3 + VO - 2H]$^{+}$	408.1327	408.1319	1.96	4	2.08
[b3 + VO - 2H]$^{+}$	436.1276	436.1270	1.38	4	2.33
[a4 + VO - 2H]$^{+}$	571.1962	571.1952	1.75	4	1.80
[b4 + VO - 2H]$^{+}$	599.1912	599.1903	1.50	4	3.35
[a5 + VO - 2H]$^{+}$	684.2804	684.2793	1.61	4	1.03
[b5 + VO - 2H]$^{+}$	712.2754	712.2744	1.40	4	0.81
[a6 + VO - 2H]$^{+}$	821.3395	821.3389	0.73	4	1.76
[a6 + VO - H]$^{2+}$	411.1734	411.1728	1.46	4	7.25
[b6 + VO - 2H]$^{+}$	849.3345	849.3328	2.00	4	12.26
[b6 + VO - H]$^{2+}$	425.1709	425.1701	1.88	4	6.90
[b7 + VO - H]$^{2+}$	473.6974	473.6966	1.69	4	1.06
[a8 + VO - H]$^{2+}$	533.2342	533.2334	1.50	4	3.00
[b8 + VO - H]$^{2+}$	547.2314	547.2309	0.91	4	2.32
[b8 + VO - 2H]$^{+}$	1093.4552	1093.4545	0.64	4	3.11
[a9 + VO - H]$^{2+}$	601.7637	601.7630	1.16	4	**31.93**
[b9 + VO - H]$^{2+}$	615.7612	615.7603	1.46	4	**18.34**
[AT1 + VO]$^{2+}$	681.3085	681.3076	1.32	4	100
C-terminal fragments					
[y4" + VO - 2H]$^{+}$	578.2061	578.2050	1.90	4	3.07
[y5" + VO - 2H]$^{+}$	715.2651	715.2641	1.40	4	0.69
[y9" + VO - H]$^{2+}$	623.7950	623.7942	1.28	4	2.26
internal fragments					
[a9y4" + VO - 3H]$^{+}$	419.1163	419.1157	1.43	4	4.56
[b9y4" + VO - 3H]$^{+}$	447.1113	447.1106	1.57	4	5.07
[a9y9" + VO - 2H]$^{2+}$	544.2502	544.2494	1.47	4	8.66
[a9z9 + VO - 2H]$^{2+}$	535.7369	535.7361	1.49	4	0.79
[b9y9" + VO - 2H]$^{2+}$	558.2478	558.2469	1.61	4	0.94

[a9y7" + VO - 3H]$^+$	832.3238	832.3220	2.16	4	0.89
[a6y7" + VO - 3H]$^+$	451.1426	451.1419	1.55	4	0.93
[b6y7" + VO - 3H]$^+$	479.1376	479.1368	1.67	4	1.24
[a9y8" + VO - 3H]$^+$	931.3915	931.3908	0.75	4	0.97
[a9y5" + VO - 3H]$^+$	556.1754	556.1745	1.62	4	1.11
[a6y9" + VO - 2H]$^{2+}$	353.6599	353.6593	1.70	4	0.58
[b6y9" + VO - 2H]$^{2+}$	367.6572	367.6568	1.09	4	0.21

References.

(1) Bühl, M.; Kabrede, H. Geometries of Transition-Metal Theory and Computation. *J. Chem. Theory Comput.* **2006**, *2* (5), 1282–1290. https://doi.org/10.1021/ct6001187.

(2) Sanna, D.; Lubinu, G.; Ugone, V.; Garribba, E. Influence of Temperature on the Equilibria of Oxidovanadium(IV) Complexes in Solution. *Dalt. Trans.* **2021**, *50* (44), 16326–16335. https://doi.org/10.1039/d1dt02680a.
